# Loss of HRD functional phenotype impedes immunotherapy and can be reversed by HDAC inhibitor in ovarian cancer

**DOI:** 10.7150/ijbs.79654

**Published:** 2023-03-21

**Authors:** Dong Yang, Fu-Xue Huang, Wei Wei, Qia-Qia Li, Jun-Wan Wu, Yun Huang, Zhi-Ling Li, Hai-Liang Zhang, Xuan Li, Qiu-Er Yuan, Qing-shan Chen, Gong-kan Feng, Deng Rong, Jun-Dong Li, Xiao-Feng Zhu

**Affiliations:** 1 State Key Laboratory of Oncology in South China, Collaborative Innovation Center for Cancer Medicine, Guangdong Key Laboratory of Nasopharyngeal Carcinoma Diagnosis and Therapy, Sun Yat-sen University Cancer Center, Guangzhou, China.; 2 Department of Gynecological Oncology, Sun Yat-sen University Cancer Center, Guangzhou, China.; 3 Department of Radiation Oncology & Therapy, Jilin Provincial Key Laboratory of Radiation Oncology & Therapy, The First Hospital of Jilin University, Changchun, China.; 4 Department of Radiation Oncology, The First Affiliated Hospital, Sun Yat-sen University, Guangzhou 510080, China

**Keywords:** Ovarian cancer, Homologous recombination deficiency, Transcriptomic functional Phenotype, Immunotherapy

## Abstract

In recent years, homologous recombination deficiency (HRD) has not achieved the expected substantial promotion of immunotherapeutic efficacy in ovarian cancer. This study aims to explore the role of HRD functional phenotype as a powerful biomarker in identifying HRD patients who may benefit from immunotherapy. HRD functional phenotype, namely HRD-EXCUTE, was defined as the average level of the 15 hub genes upregulated in HRD ovarian cancer. A decision tree was plotted to evaluate the critical role of HRD-EXCUTE in HRD patients. Agents inducing HRD-EXCUTE were identified by CMAP web (Connectivity Map). The mechanisms and immunotherapeutic effect of PARPi and HDACi in promoting HRD-EXCUTE was examined *in vitro* and *in vivo*. The decision tree plotted on the basis of HRD and HRD-EXCUTE indicated the HRD patients without the HRD functional phenotype were largely unresponsive to immunotherapy, which was validated by the immunotherapeutic cohorts. Furthermore, loss of HRD-EXCUTE in the HRD patients attenuated immunogenicity and inhibited immune cells in tumor microenvironment. Moreover, Niraparib combined with Entinostat induced HRD-EXCUTE by activating the cGAS-STING pathway and increasing the histone acetylation. The combination therapy could enhance the cytotoxicity of immune cells, and promote pro-immune cells infiltrating into ascites, resulting in inhibited ovarian cancer growth. The HRD functional phenotype HRD-EXCUTE was set up as a potent biomarker to identify whether HRD patients can benefit from immunotherapy. Loss of HRD-EXCUTE in HRD patients were largely insensitive to immunotherapy. The combination of PARPi with HDACi could improve the efficacy of the PARPi-based immunotherapy in ovarian cancer by augmenting the HRD functional phenotype.

## Introduction

Cancer with homologous recombination deficiency (HRD), particularly ovarian cancer, preserves basal genomic stability by alternative DNA damage repair (DDR) mechanisms, making HRD cancer cells susceptible to DNA damage therapies such as poly (ADP-ribose) polymerase inhibitors (PARPi).^1^-^3^ A mass of aberrant endogenous DNA fragments produced by HRD tumors can activate innate immunity by the cGAS-STING pathway, etc.^4^ Collectively, HRD is a potential biomarker for DNA damage therapy and immunotherapy.

There have been many evaluation systems established for the identification of HRD positive patients, including gene mutations in the DDR pathway (BRCAness, etc.) and genomic scars.^5^ BRCAness stands for cancer cells with molecular characteristics similar to BRCA-mutant cells,^6^ while genomic scars is recognized as another promising detection method which is based on estimating HRD levels from specific DNA aberrations, such as HRD Score, HRDetect.^5^ HRD Score, approved by FDA as an efficient biomarker for first-line olaparib plus bevacizumab-based therapy in ovarian cancer^7^, incorporates loss of heterozygosity (LOH), telomeric allelic imbalance (TAI), and large-scale state transitions (LST)^5^.

Theoretically, it should be rational to apply immunotherapies in ovarian cancers, since approximately 50% of ovarian cancers harbor HRD.^8^ However, the results of clinical trials indicated that patients with ovarian cancer could not benefit from immunotherapies as expected. The objective response rate (ORR) of single immune checkpoint blockade (ICB) ranged from 6% to 22%, regardless of HRD status.^9^-^11^ Furthermore, ICB-PARPi combined therapies have been proposed to promote immunotherapeutic efficacy in ovarian cancer.^12^,^13^ However, it failed to achieve the expected efficacy regardless of various HRD levels.^14^,^15^ The reasons why HRD failed to be an expected effective biomarker for ovarian cancer immunotherapy remain elusive. It is necessary to find a potent biomarker to further distinguish the applicability of immunotherapies for HRD patients. The transcriptome is an essential mediator of genomic regulation of cellular functional phenotypes. Considering that some HRD patients might lack immunogenicity in functional phenotype,^16^ we hypothesized that transcriptomic functional status could be utilized to determine immunogenicity and prognosis of HRD patients.

Here, we implemented a transcriptional profiling-based approach to systematically identify the functional status of HRD positive cancers. By analyzing the differential expression patterns of HRD positive and negative patients in TCGA-OV cohort, we found that 15 hub genes could model the immune functional phenotype in ovarian cancer. The combined pattern of these hub genes was termed as HRD-EXCUTE. Survival decision tree further revealed that HRD patients who gained HRD-EXCTUE had substantially prolonged survival, and HRD patients without HRD-EXCUTE had worse prognosis which was similar to HRD negative patients, as confirmed by other cohorts and two immunotherapy trials. Mechanistically, HRD-EXCUTE prompts higher tumor mutation burden and more neoantigens to inflame tumor microenvironment, significantly boosting B cells, CD4^+^ T cells, CD8^+^ T cells, etc. Moreover, we found that the HDAC deacetylation was significantly activated in HRD patients without HRD-EXCUTE. The combination of HDAC inhibitor (HDACi) Entinostat and PARPi could significantly promote HRD-EXCUTE by activating the cGAS-STING pathway and increasing histone acetylation around genomic locus of HRD-EXUTE hub genes. The combination strengthened PARPi-based immunotherapy in HRD ovarian cancer mouse model. In summary, we identified a transcriptomic functional phenotype of HRD which indicates stronger immunogenicity of tumors, and distinguished HRD patients who had better prognosis. We recommend a therapy based on the combination of PARPi and HDACi to improve the efficacy of PARPi-based immunotherapy.

## Results

### HRD promoted immune-transcriptomic phenotype in ovarian cancer

**Figure 1:**
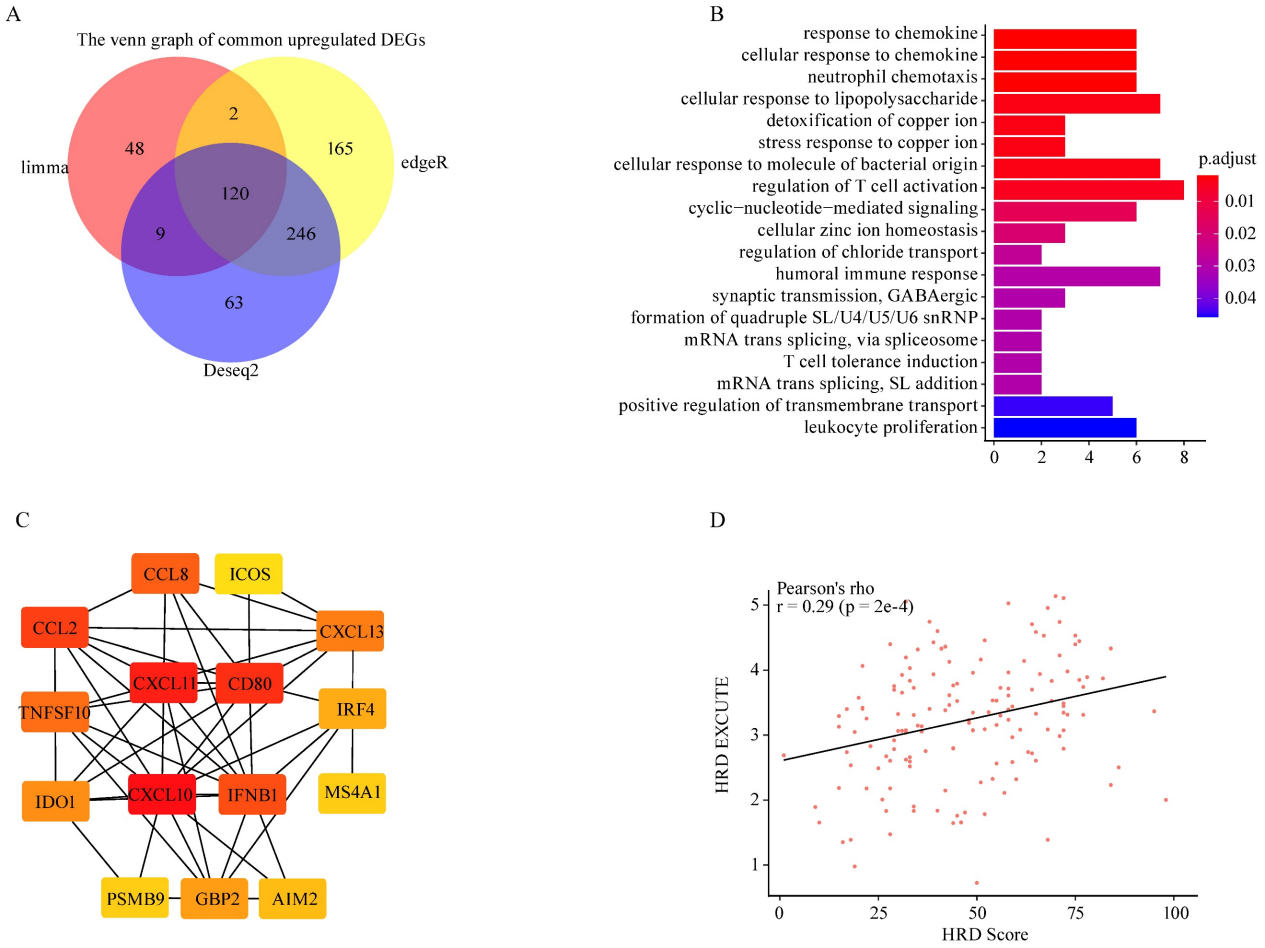
** HRD promoted immune-transcriptomic phenotype in ovarian cancer**. (A) Venn map of common upregulated genes in HRD-high group among *Limma*, *EdgeR* and *Deseq2* methods. (B) The GO biological process enrichment of upregulated genes in HRD-high group. (C) The correlation network of 15 hub genes generated by STRING and Cytoscape app. The correlation degree increased as the box color turned from red to yellow. (D) The scatter was plotted to present the correlation between HRD and HRD-EXCUTE.

To explore HRD transcriptomic functional phenotype, three methods (*Limma, EdgeR, DESeq2*) were applied to determine the differences in gene expression between HRD high and low group based on TCGA-OV dataset. The two different HRD groups were separately defined as top 20% (HRD high) and bottom 20% (HRD low) of HRD Scores. A total of 266 common differently expressed genes were screened out, of which 120 genes were upregulated and 146 genes were downregulated (**Fig. 1A**; Supplementary Fig. S1A-B). The upregulated parts were significantly enriched in the immune-related pathways where the chemokine response was mostly enriched (**Fig. 1B**). The downregulated parts were mainly enriched in the hydrogen peroxide related pathways, etc. (Supplementary Fig. S1C). Protein-protein interactions (PPI) of upregulated genes was analyzed on the *STRING* website, and the *Cytoscape* was utilized to figure out the hub genes from the PPI network. Fifteen genes were determined as the hub genes in the upregulated parts in the HRD high group, including *CXCL10*,* CXCL11*,* CD80*,* IFNB1*,* CXCL13*,* CCL2*,* CCL8*,* TNFSF10*,* IDO1*,* IRF4*,* GBP2*,* AIM2*, *PSMB9*,* MS4A1*,* ICOS* (**Fig. 1C**). The 15 hub genes presented constant expression levels in ovarian tumor (Supplementary Fig. S1D). HRD transcriptomic functional phenotype was then characterized by the average expression of the 15 hub genes, namely HRD-EXCUTE which was positively correlated with HRD Score (**Fig. 1D**). Together, HRD profoundly remodeled the tumor immune-landscape and turned “cold” tumor to the “hot”, which was tallied with the speculation in previous studies.^21^

### HRD transcriptomic phenotype potently determined the prognosis in HRD patients

**Figure 2:**
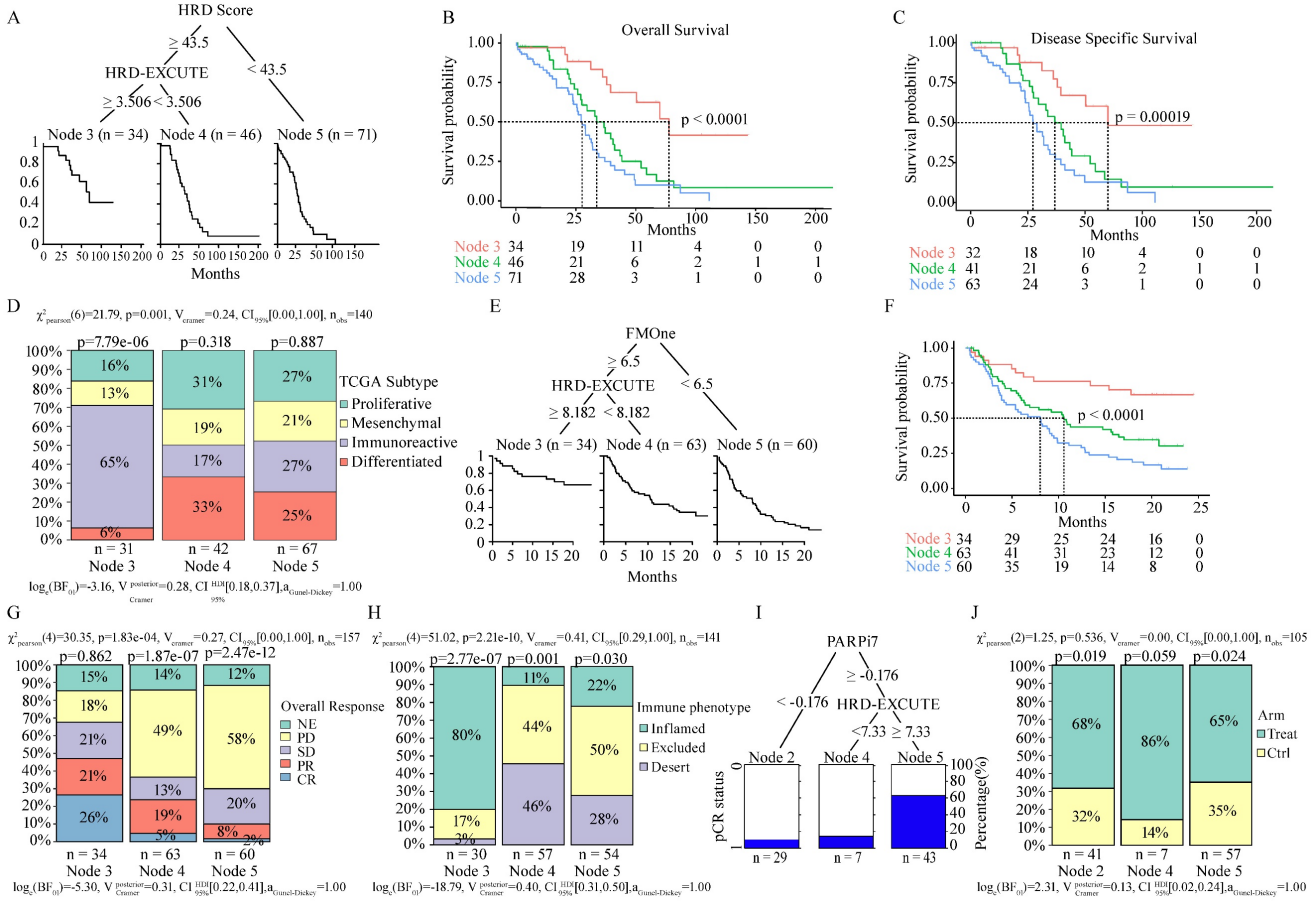
** HRD-EXCUTE determined HRD patients' prognosis and immunotherapeutic efficacy**. (A) The decision tree of HRD and HRD-EXCUTE in TCGA-OV. In the tree, Node 3: HRD^+^HRD-EXCUTE^+^; Node 3: HRD^+^HRD-EXCUTE^-^; Node 5: HRD^-^. (B) The overall survival, (C) the disease specific survival in three subgroups of the decision tree in TCGA-OV. (D) The distribution of immune phenotypes among three subgroups of the decision tree in TCGA-OV. (E) The decision tree of FMOne and HRD-EXCUTE in IMvigor210 cohort. In the tree, Node 3: FMOne^+^HRD-EXCUTE^+^; Node 3: FMOne^+^HRD-EXCUTE^-^; Node 5: FMOne^-^. (F) The overall survival in three subgroups of the decision tree in IMvigor210 cohort. The plotted percentage stacked bar represented (G) the overall response and (H) the immune phenotypes in the three subgroups of the decision tree in IMvigor210 cohort. (I) The decision tree of PARPi7 and HRD-EXCUTE in I-SPY2 trial. In the tree, Node 2: PARPi7^-^; Node 4: PARPi7^+^HRD-EXCUTE^-^; Node 5: PARPi7^+^HRD-EXCUTE^+^. The plotted percentage stacked bar showed (J) the distribution of treatment group in three subgroups of the decision tree in I-SPY2 trial.

To investigate whether HRD transcriptomic functional phenotype could predict the prognosis of HRD patients, dichotomous decision tree was applied to analyze the prognostic significance of HRD-EXCUTE in HRD ovarian cancer. In the resulting tree, ovarian cancer patients were divided into three subgroups termed as HRD^+^HRD-EXCUTE^+^(Node3), HRD^+^HRD-EXCUTE^-^(Node4) and HRD^-^(Node5) (**Fig. 2A**). The results of Kaplan-Meier (KM) survival analysis indicated that the HRD^+^HRD-EXCUTE^+^ patients had better overall survival, Disease-free survival and progress-free survival, compared to other two subgroups (**Fig. 2B-C**; Supplementary Fig. S2A). Furthermore, the complete remission rate of the HRD^+^HRD-EXCUTE^+^ subgroup was 86%, which was higher than that of other two subgroups, though the difference was not statistically significant (Supplementary Fig. S2B). In the analysis of tumor microenvironmental subtypes, HRD^+^HRD-EXCUTE^+^ subgroup had more immune-inflamed subtypes than other two subgroups according to the results by different methods (**Fig. 2D**; Supplementary Fig. S2C-D). HRD^+^HRD-EXCUTE^-^ and HRD^-^ subgroup trended to possess proliferative, differentiated and mesenchymal subtypes (**Fig. 2D**).

Next, we integrated 10 Affymetrix transcriptomic arrays (affy cohort) of ovarian cancer to further confirm HRD-EXCUTE function in the prognosis of HRD patients (Supplementary Fig. S2E-F). Due to the lack of HRD score in affy cohort, we applied the Repair Proficiency Score (RPS) to estimate the efficiency of DNA repair. Low RPS positively correlated with HRD and the sensitivity to DNA damage-inducing agents.^22^ Affy cohort was divided into three groups according to the median of RPS and HRD-EXCUTE, and RPS^-^HRD-EXCUTE^+^ subgroup had the best overall survival among three groups in the affy cohort (Supplementary Fig. S2G). In the TCGA-LUSC/CESC datasets, HRD-EXCUTE could also significantly discriminate patients with good prognosis from HRD positive group (Supplementary Fig. S2H-I). In summary, patients in HRD^+^HRD-EXCUTE^-^ and HRD^-^ subgroup had worse prognosis and more aggressive cancer subtypes, compared to patients in HRD^+^HRD-EXCUTE^+^ subgroup, which indicated that the HRD-EXCUTE, as a HRD transcriptomic functional phenotype, could potently predict the prognosis of HRD ovarian cancer patients.

### HRD transcriptomic phenotype determined the immunotherapeutic efficacy

We then investigated whether HRD-EXCUTE could predict the immunotherapeutic efficacy of HRD patients. Due to the lack of published omics of ovarian cancer patients who received immunotherapy, the well-studied immunotherapeutic cohorts of two different cancers (urothelial cancer and breast cancer) were selected as research subjects, HRD patients in which benefited from immunotherapy.^23^,^24^ Clinical efficacy of patients with metastatic urothelial cancer who received anti-PD-L1 treatment (atezolizumab) were examined by IMvigor210 cohort.^17^ Due to the lack of HRD Score testing, IMvigor210 cohort instead assessed the FMOne mutation burden, which was also an indicator of HRD.^17^ As shown in **Fig. 2E-F**, the cohort could be divided in to three subgroups: FMOne^+^HRD-EXCUTE^+^ (Node 3), FMOne^+^HRD-EXCUTE^-^ (Node 4), and FMOne^-^ (Node 5) by FMOne and HRD-EXCUTE, which was similar to the situation in the TCGA-OV cohort (**Fig. 2A-B**). Patients in FMOne^+^HRD-EXCUTE^+^ subgroup (Node 3) achieved the best overall survival and response to immunotherapy among three subgroups (**Fig. 2F-G**). Approximately 80% of patients in the FMOne^+^HRD-EXCUTE^+^ subgroup were immune-inflamed, which was substantially higher than that in FMOne^+^HRD-EXCUTE^-^ or FMOne^-^ subgroup (**Fig. 2H**).

Next, the phase II I-SPY2 trial which aimed at treating high-risk HER2-negative stage II/III breast cancer by durvalumab combined with Olaparib and paclitaxel was utilized to further confirm the prognostic value of HRD-EXCUTE in the HRD patients.^18^ PARPi7 signature was constituted of 7 DNA repair genes (*BRCA1*,* CHEK2*,* MAPKAPK2*,* MRE11A*,* NBN*,* TDG*,* XPA*), for predicting PARP inhibitor sensitivity,^25^ and it was significantly correlated with HRD Score (Supplementary Fig.S2J). As indicated in **Fig. 2I**, PARPi7 and HRD-EXCUTE together could also divide the cohort into three subgroups: PARPi7^-^ (Node 2), PAPRi7^+^HRD-EXCUTE^-^ (Node 4), and PAPRi7^+^HRD-EXCUTE^+^ (Node 5). The treatment distribution of the three subgroups was not significantly different (**Fig. 2J**). Together, HRD-EXCUTE displayed a significant positive-correlation with immune-inflamed subtype and could substantially improve the immunotherapeutic efficacy.

### HRD transcriptomic phenotype promoted tumor immunogenicity of ovarian cancer

**Figure 3:**
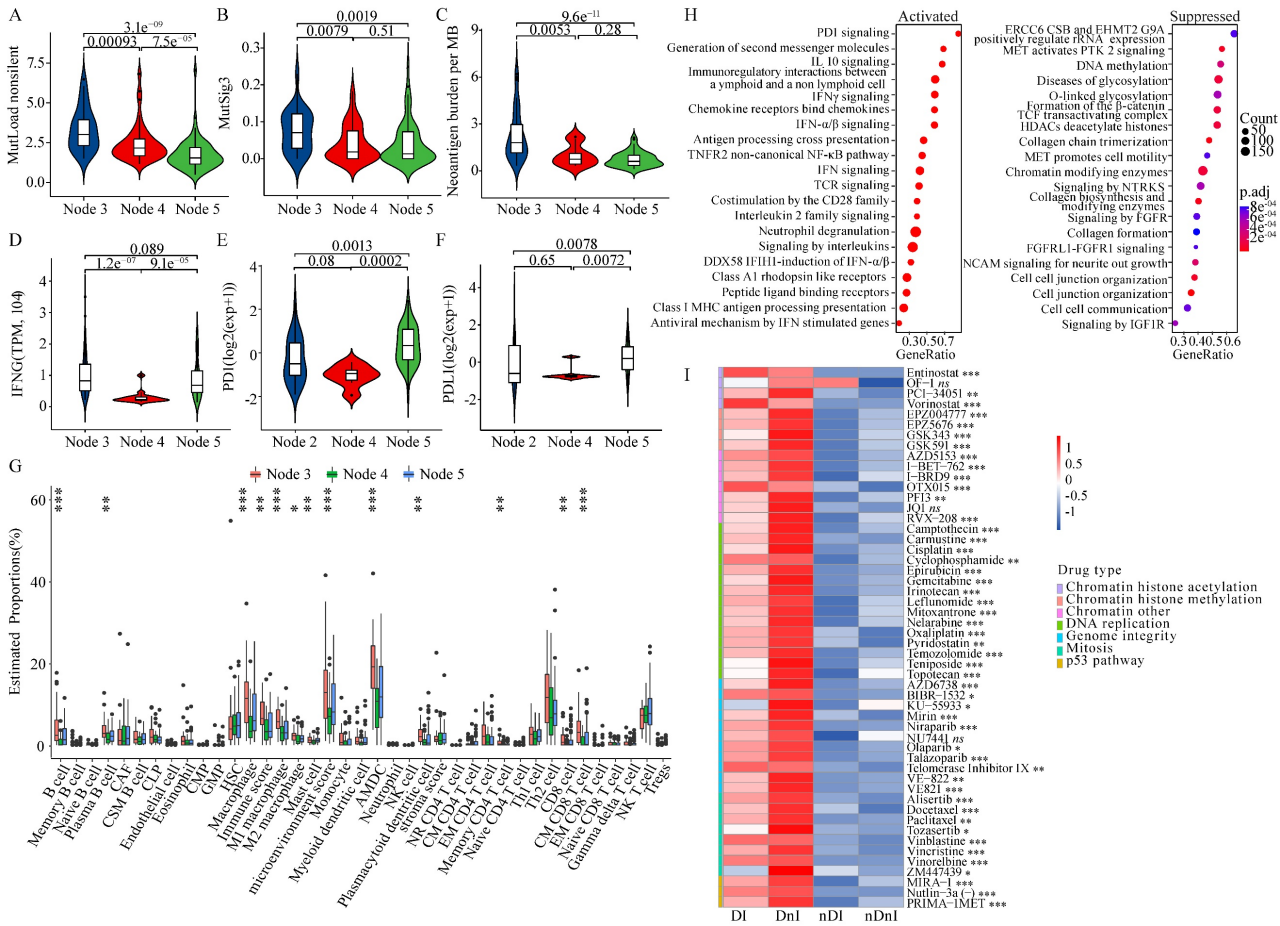
** HRD transcriptomic phenotype promoted tumor immunogenicity of ovarian cancer**. (A) the non-silent mutation load per Mb, (B) the MutSig3 from COSMIC (https://cancer.sanger.ac.uk/cosmic) in the TCGA-OV, and (C) the Neoantigen burden per MB in the IMvigor210 cohort. (D) the IFNG mRNA levels in the IMvigor210 cohort, (E) the PD-1 and (F) the PD-L1 mRNA levels in the I-SPY2 trial. The boxplots showed the tumor microenvironmental components in (G) the TCGA-OV. The microenvironmental components were estimated by *xCell* algorithm. (H) The enrichment plots of the differential genes between HRD^+^HRD-EXCUTE^+^ and HRD^+^HRD-EXCUTE^-^ subgroups in the TCGA-OV cohort. The left part indicated upregulated pathways in HRD^+^HRD-EXCUTE^+^ subgroup and the right part indicated upregulated pathways in HRD^+^HRD-EXCUTE^-^ subgroup. (I) The heatmap showed the log (IC50) levels of DNA damage agents among four subgroups of CCLE cell lines. DI: HRD^+^HRD-EXCUTE^+^; DnI: HRD^+^HRD-EXCUTE^-^; nDI: HRD^-^HRD-EXCUTE^+^; nDnI: HRD^-^HRD-EXCUTE^-^.

We aimed to figure out whether HRD-EXCUTE was related to tumor immunogenicity. We found that the HRD^+^HRD-EXCUTE^+^ subgroup had the most mutation burden among the three subgroups (**Fig. 3A**; Supplementary Fig. S3A) and several mutational signatures were significantly upregulated in the HRD^+^HRD-EXCUTE^+^ subgroup (**Fig. 3B**; Supplementary Fig. S3B-C). In the IMvigor210 cohort, the FMOne^+^HRD-EXCUTE^+^ subgroup possessed more tumor neoantigens than any other subgroups (**Fig. 3C**). The IFNG expression was the most significantly upregulated in the FMOne^+^HRD-EXCUTE^+^ subgroup (**Fig. 3D**). Additionally, the PD-1-PDL1 expression in PAPRi7^+^HRD-EXCUTE^+^ subgroup was significantly higher than other two subgroups in the I-SPY2 trial (**Fig. 3E-F**).

We then investigated the differences in cell proportions of tumor microenvironment among three subtypes in the three cohorts (**Fig. 3G**; supplementary Fig. S3D-E). Altogether, the immune score and microenvironment score of HRD^+^HRD-EXCUTE^+^ subgroup were the highest among three subgroups. In details, HRD^+^HRD-EXCUTE^+^ subgroup had significantly higher proportions of B cells, plasma B cells, M1 macrophages, Activated myeloid dendritic cells, plasmacytoid dendritic cells, memory CD4^+^ T cells, Th2 cells, CD8^+^ T cells, central memory CD8^+^ T cells, effector memory CD8^+^ T cells, gamma delta T cells and regulatory T cells. Together, we concluded that HRD-EXCUTE could increase tumor immunogenicity by upregulating tumor mutation burden and neoantigen quantity, promoting immune cell infiltration, and remodeling the immune-inflamed microenvironment.

We further explored whether the favorable prognosis of patients in the HRD^+^HRD-EXCUTE^+^ subgroup were mainly associated with the immunological program. As showed in the **Fig. 3H**, more immune-related pathways clustered in the HRD^+^HRD-EXCUTE^+^ subgroup, such as PD-1 signaling and IFNγ signaling, while more growth signaling were enriched in HRD^+^HRD-EXCUTE^-^ subgroup, such as MET signaling and signaling by FGFR. The CCLE cell lines were divided into four cancer cell subgroups according to the median expression levels of HRD Score and HRD-EXCUTE. As indicated in the **Fig. 3I**, the cancer cell lines in HRD^+^ groups were more sensitive to DNA damage agents than HRD^-^ groups, approving that HRD was the sensitive biomarker for DNA damage agents. But the cell lines in HRD^+^HRD-EXCUTE^+^ group were more resistant to most of DNA damage agents than the cells in HRD^+^HRD-EXCUTE^-^ group without statistical significance. The expression levels of PARP9/10/14 were significantly upregulated in the HRD^+^HRD-EXCUTE^+^ group (Supplementary Fig. S3F), which might be the resistance factors against DNA damage agents in HRD^+^HRD-EXCUTE^+^ group. Moreover, the transcriptomic phenotype of cell lines in HRD^+^HRD-EXCUTE^+^ group were more immune-inflamed than that in HRD^+^HRD-EXCUTE^-^ group (Supplementary Fig. S3G). Altogether, HRD-EXCUTE could not increase drug sensitivity of HRD cancer cells, but mainly promoted the immunological phenotype in HRD cells.

### The combination of HDACi and PARPi greatly promoted HRD-EXCUTE

**Figure 4:**
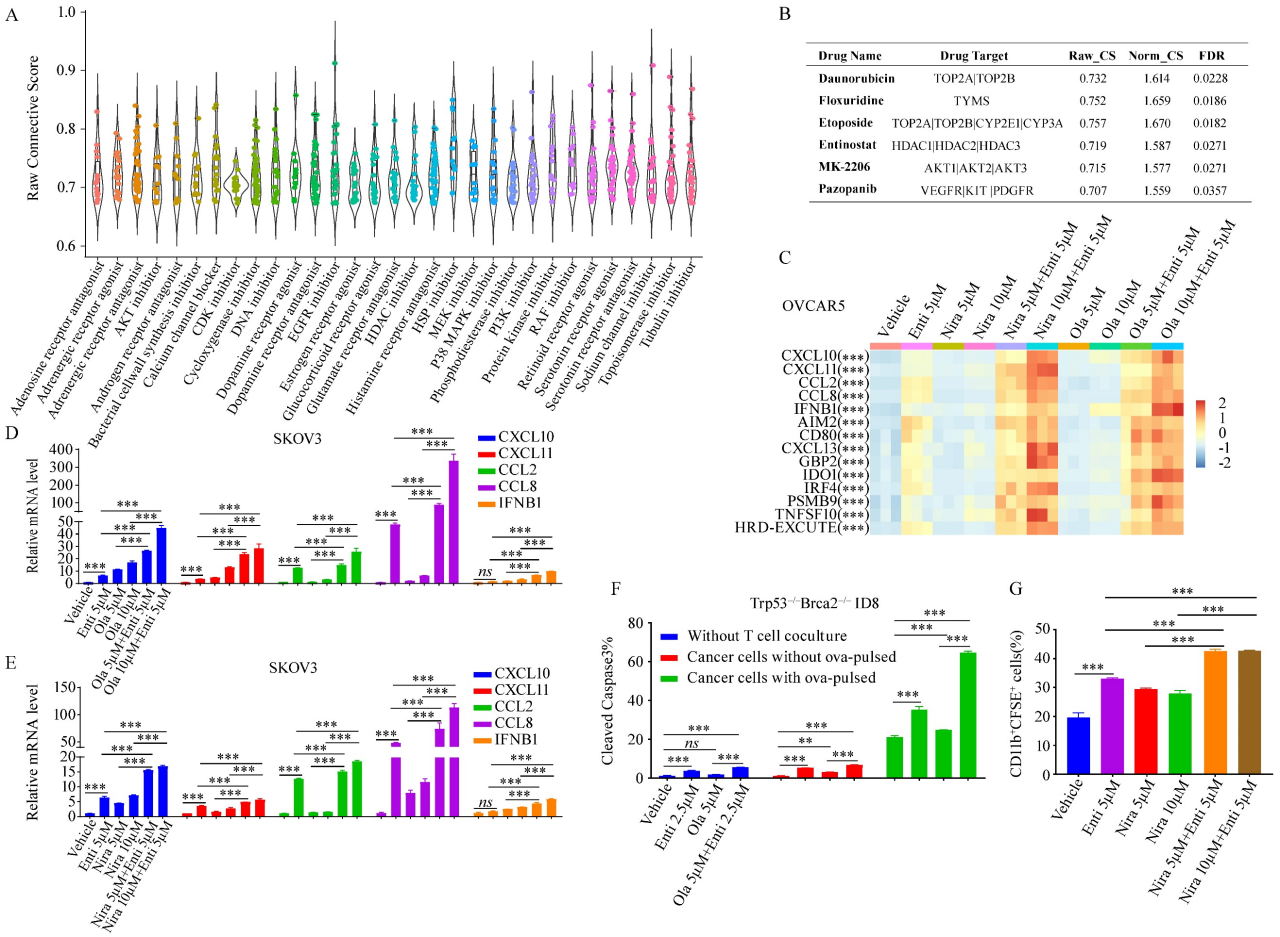
** The combination of HDACi and PARPi greatly promoted HRD-EXCUTE.** (A) The boxplot demonstrated the raw connective scores of agents inducing HRD-EXCUTE, and the horizontal axis showed the agent types. Each dot in the plot represented one agent. (B) The table showed the detailed parameters for the four drugs in the CAMP result. Raw_CS: raw connective score, Norm_CS: normalized connective score. The relative expression levels of CXCL10/11, CCL2/8 and IFNB1 were shown in C-F. (C) Heatmap showed the relative expression levels of HRD-EXCUTE hub genes in OVCAR5. Expression levels were scaled over all groups. SKOV3 cells were treated by (D) Olaparib, (E) Niraparib and Entinostat for 12 hours, followed by qPCR to examine the expression levels of HRD-EXCUTE hub genes. (F) Boxplot showed the percentages of cleaved caspase-3 positive cells in Trp53^-/-^Brca2^-/-^-ID8 cells. The cells were pre-treated by Olaparib and Entinostat, followed by co-culturing with activated OT-1 T cells for 3 hours. (G) Boxplot showed the percentage of CD11b^+^CFSE^+^ cells in phagocytosis assay. The cells were pre-treated by Olaparib and Entinostat and labeled by CFSE, followed by co-culturing with activated BMDM for 2 hours.

Since HRD-EXCUTE could effectively define the immunotherapeutic efficacy of HRD patients, we hypothesized that promoting HRD-EXCUTE could improve prognosis of the HRD patients. In order to screen out agents that could induce the HRD-EXCUTE signature, we compared and analyzed data from the *CMAP* web with the HRD-EXCUTE signature, aiming to find out drugs which possess gene expression signatures overlapped with the HRD-EXCUTE signature, and this kind of drugs might have potential to induce the sensitivity towards immunotherapy. Remarkably, we found that most of DNA damage drugs were in the top-ranking of agents that can induce the HRD-EXCUTE-like signatures, which was consistent with the fact that HRD could promote HRD-EXCUTE (**Fig. 4A-B**). The topoisomerase II inhibitor Etoposide could significantly promote the expression levels of CXCL10/11, CCL2/8 and IFNB1 in the HRD-EXCUTE (Supplementary Fig. S3A-B). We also found that many reported pro-immunogenic inhibitors could activate the expression profiles similar to HRD-EXCUTE signature, such as inhibitors of PI3K-AKT pathway, MAPK pathway and histone deacetylase (HDAC) (**Fig. 4A-B**).^26^,^27^ The AKT inhibitor MK2206 and HDAC inhibitor Entinostat could also significantly stimulate the expressions of CXCL10/11, CCL2/8 and IFNB1 in the HRD-EXCUTE (Supplementary Fig. S4C-D).

PARP inhibitors have the potential to enhance immunotherapy via promoting tumor immunogenicity by cGAS-STING pathway.^28^ But PARPi-based immunotherapies did not function for HRD patients as efficiently as expected, which indicated the existence of intrinsic resistant factors in HRD tumor to impede the enhancement of immunogenicity by PARPi. Moreover, HDAC deacetylation was significantly downregulated in HRD-EXCUTE^+^ subgroup and upregulated in HRD-EXCUTE^-^ subgroup in HRD patients (**Fig. 3H**). HDAC inhibitor Entinostat significantly promoted HRD-EXCUTE signature (Supplementary Fig. S4D), which could potentially enhance immunogenicity in HRD tumors. Here, we speculated that PARPi could efficiently promote immunogenicity in HRD tumors when combined with HDACi. We found that PARP inhibitors Niraparib and Olaparib were unable to significantly promote HRD-EXCUTE signature, but the combination of PARPi and the selective HDAC1/3 inhibitor Entinostat further improved HRD-EXCUTE in ovarian cancer (**Fig. 4C-E**; Supplementary Fig. S4E-F). In addition, Olaparib/Niraparib-Entinostat combination also upregulated HRD-EXCUTE in the normal immortalized ovarian epithelial cells (Supplementary Fig. S4G). Furthermore, tumor cells pre-treated with PARPi and HDACi were potently killed by activated CD8^+^T cells (**Fig. 4F**). Moreover, tumor cells pre-treated with PARPi and HDACi were also significantly phagocytized by the activated macrophages (**Fig. 4G**). Together, we concluded that PARPi combined with HDACi could significantly promote HRD-EXCUTE signature to enhance immunogenicity.

**Figure 5:**
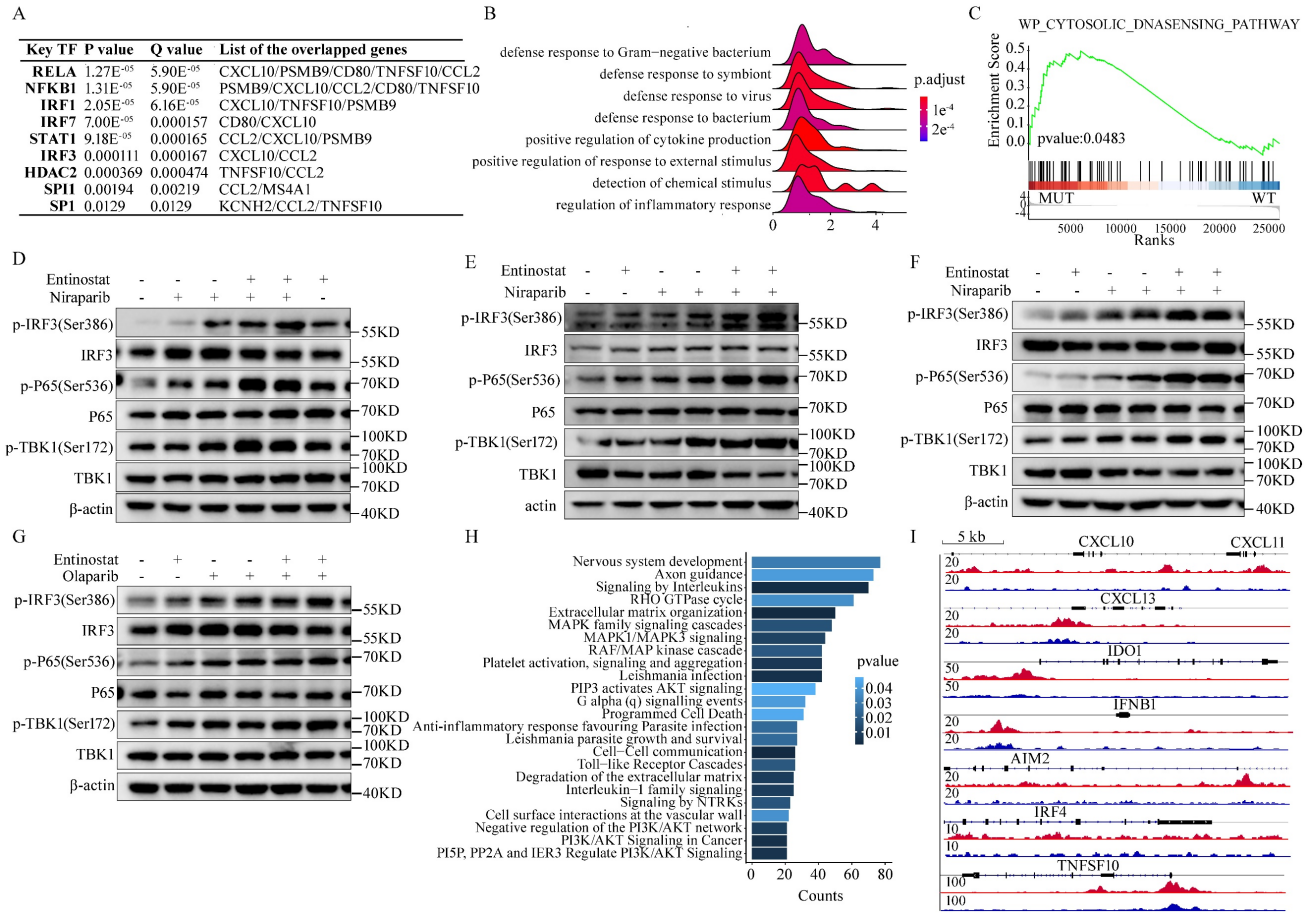
** Mechanisms of the combination of PARPi and HDACi in stimulating HRD-EXCUTE**. (A) The table showed the predicted transcriptional factors of HRD-EXCUTE in TRUSST web. (B) Ridge plot of GO biological progress and (C) GSEA plot showed the upregulated pathways in BRCA1-null ovarian cancer cells under the treatment of Entinostat in the GSE131085 dataset; (D)SKOV3, (E) OVCAR5, (F)CAOV3 cells were treated with Niraparib (5,10μM) and Entinostat (5μM) for 12 hours, and (G) OVCAR5 cells were treated with Olaparib (5,10μM) and Entinostat (5μM) for 12 hours. The proteins were extracted for western blotting to examine the phosphorylation of TBK1, IRF3 and P65. Chip-seq data from (H, J) GSE131140 dataset were applied to analyze the H3K27ac acetylation levels of HRD-EXCUTE hub genes: (H) Enriched Reactome pathways in the BRCA1-null ovarian cancer cells; (I) IGV map showed the H3K27ac peaks around genomic locus of HRD-EXCUTE genes. Red: BRCA1-null cells, and blue: BRCA1-wt cells.

Next, we explored the possible mechanism behind the induction of the HRD-EXCUTE signature by the combination of PARPi and HDACi. Firstly, the *TRRUST* database was applied to find out the potent transcriptional factors that can stimulate HRD-EXCUTE.^29^ It turned out that the NF-κB and IRF signaling were the most significantly predicted potent transcriptional factors of HRD-EXCUTE (**Fig. 5A**). The NF-κB and IRF3 signaling were the main following cascade in the cGAS-STING pathway.^30^ Entinostat efficiently activated immune-related pathways (**Fig. 5B**) and cytosolic DNA sensing pathway (**Fig. 5C**) in BRCA1-null ovarian tumor cells. Furthermore, the phosphorylation levels of TBK1, P65, and IRF3 were significantly elevated in ovarian cancer cells and normal immortalized ovarian epithelial cells treated by both PARPi and Entinostat, indicating the activation of cGAS-STING pathway (**Fig. 5D-G**, Supplementary Fig. S5A). Moreover, our RNA sequencing results showed that OVCAR5 cells treated with PARPi-Entinostat combination were significantly distinctive from those treated with a single agent (Supplementary Fig. S5B). The hub genes in the HRD-EXCUTE were significantly upregulated in the PARPi-Entinostat treatment (Supplementary Fig. S5C). The functional enrichment analysis showed that the PARPi-Entinostat treatment could greatly promote immune-related pathways, such as the response to interferon-gamma in the Niraparib-Entinostat therapy, and the regulation of leukocyte migration in the Olaparib-Entinostat treatment (Supplementary Fig. S5D). The PARPi-Entinostat treatment could also suppress several pathways involved in genome stability and RNA processing, which may increase tumor immunogenicity (Supplementary Fig. S5E). Furthermore, Niraparib-Entinostat treatment could activate the Cytosolic DNA sensing pathway (Supplementary Fig. S5F). Altogether, Our RNA-seq results also demonstrated that the PARPi-HDACi treatment could significantly promote the HRD-EXCUTE and tumor immunogenicity.

In addition, higher H3K27ac levels were detected on genes related to signaling of interleukins in BRCA1-null tumor cells (**Fig. 5H**). More H3K27ac clustered around the genomic locus of the HRD-EXCUTE hub genes in BRCA1-null ovarian cancer cells (**Fig. 5I**). It indicated that the histone acetylation was the key factor of activating HRD-EXCUTE. In conclusion, HDAC inhibitor Entinostat could elevate histone acetylation around HRD-EXCUTE hub genes and promote the cGAS-STING pathway by combining with PARPi, which substantially promoted HRD-EXCUTE signature.

### PARPi-HDACi combined treatment improved tumor immunotherapy in HRD tumor

**Figure 6:**
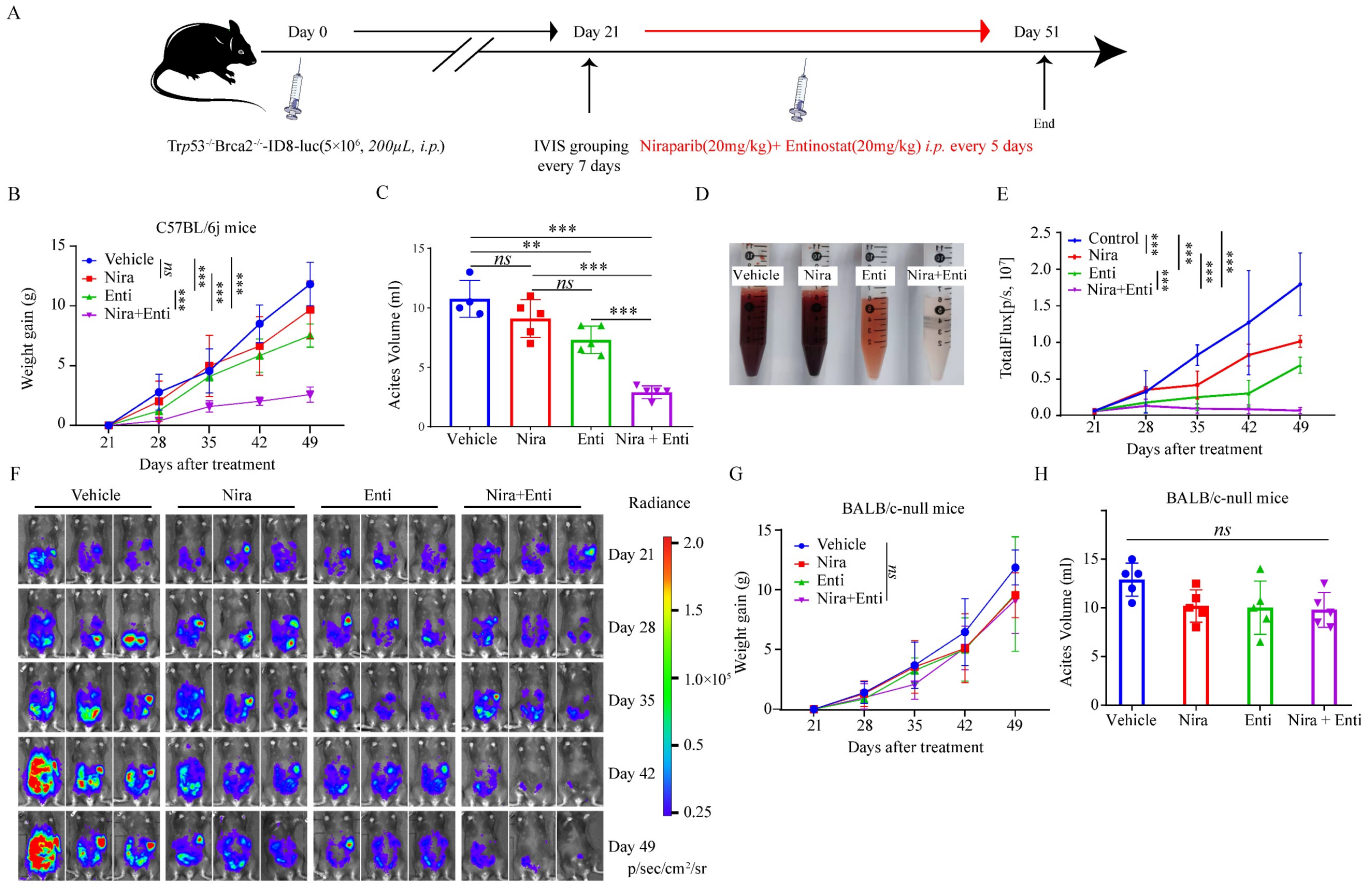
** Niraparib combined with Entinostat suppressed HRD ovarian tumor growth *in vivo*.** (A) Diagram illustrated the processes of animal experiment. (B) The tumor burden was evaluated by weighing weekly after treatment. Data were presented by mean value of 5 mice per group ± standard error. (C) Bar plot showed ascites volume (ml) by the end of experiment in four subgroups. (D)The picture showed the bloody degree of ascites in four subgroups. A volume of 6ml ascites in each subgroup was collected for examination. (E) The tumor burden was evaluated by quantifying total flux with PE Living Image software. Data were presented by mean value of 5 mice per group ± standard error. (F) Bioluminescence of C57BL/6j mice after inoculation of Trp53^-/-^Brca2^-/-^-ID8-Luc cells. (G) The tumor burden was evaluated by weighing weekly after treatment. Data were presented by mean value of 5 mice per group ± standard error. (H) Bar plot showed ascites volume (ml) by the end of experiment in four subgroups. Nira: Niraparib, Enti: Entinostat.

**Figure 7:**
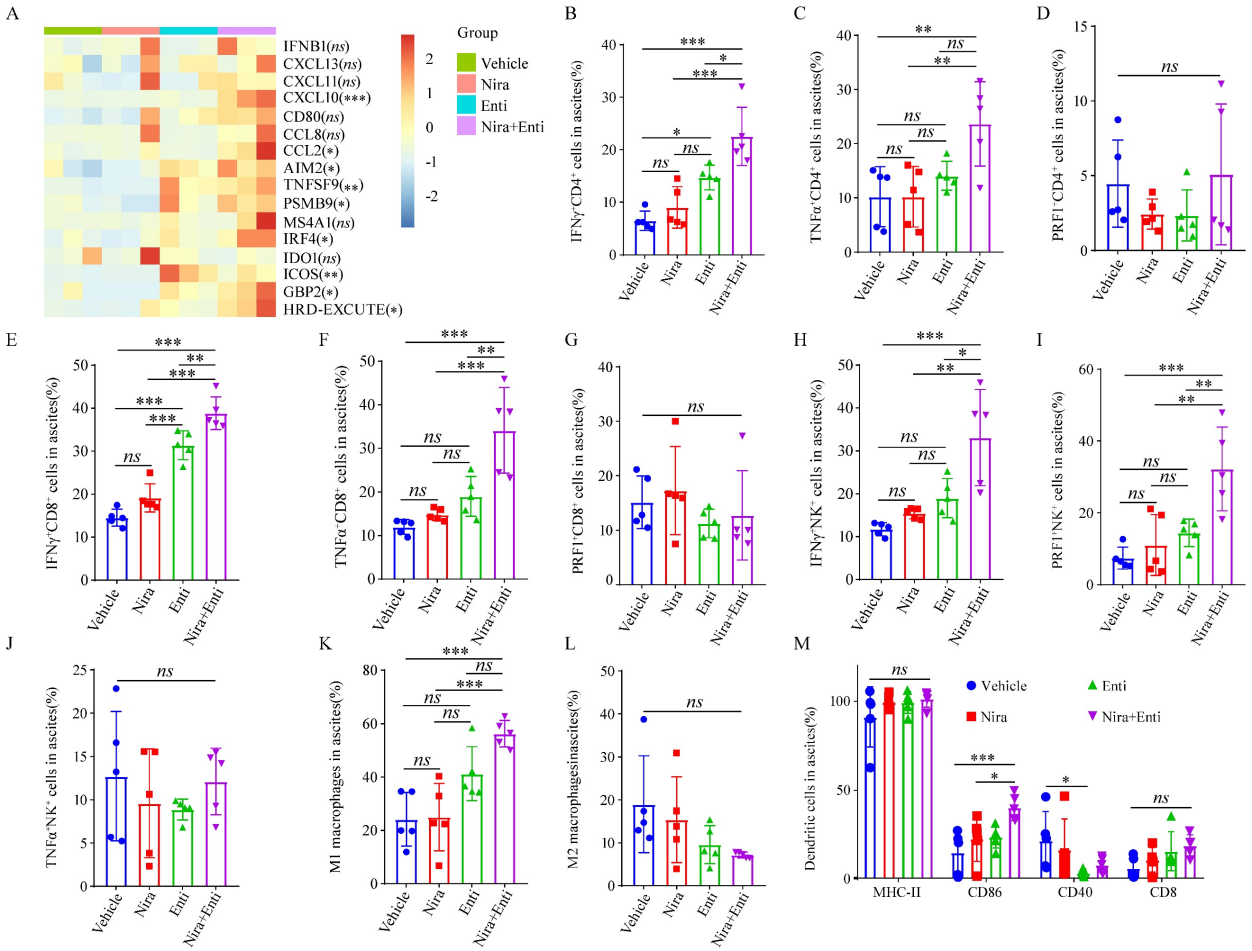
** Double combination therapy promoted HRD-EXCUTE and facilitated lymphocytes infiltrating in ascites.** (A) Heatmap showed the relative expression levels of HRD-EXCUTE hub genes in ascites cells. Boxplots showed the fraction of immune cells: (B) IFNγ^+^CD4^+^T cells, (C) TNFα^+^CD4^+^T cells, (D) PRF1^+^CD4^+^T cells, (E) IFNγ^+^CD8^+^T cells, (F) TNFα^+^CD8^+^T cells, (G) PRF1^+^CD8^+^T cells, (H) IFNγ^+^NK cells, (I) TNFα^+^NK cells, (J) PRF1^+^NK cells, (K) M1 macrophages, (L) M2 macrophages and (M) DC cells. Nira: Niraparib, Enti: Entinostat.

Finally, we explored the immunotherapeutic efficacy of PARPi-HDACi combined therapy *in vivo*. Trp53^-/-^Brca2^-/-^-ID8-Luc cells were utilized to build up HRD tumor model. The diagram of animal experiment was shown in **Fig. 6A**. Combination of Niraparib and Entinostat could significantly delay severe ascites occurring, while neither Entinostat alone nor Niraparib alone had such effect (**Fig. 6B-C**). Moreover, the combination therapy also reduced bloody ascites (**Fig. 6D**). Either Niraparib or Entinostat could inhibit tumor growth, but the combination therapy achieved much better inhibition effect of tumor growth (**Fig. 6E-F**). Furthermore, the combination therapy significantly promoted HRD-EXCUTE signature of ascites cells (**Fig. 7A**) and upregulated the number of immune cells in ascites, which consequently remodeled tumor microenvironment in ascites (**Fig. 7B-M**). In details, the amounts of IFNγ^+^CD4^+^T cells, TNFα^+^CD4^+^T cells, IFNγ^+^CD8^+^T cells, TNFα^+^CD8^+^T cells, IFNγ^+^NK cells, PRF1^+^NK cells, M1 macrophages and CD86^+^ DC cells in ascites were significantly upregulated in the combination group. The ratio of ascitic M2 macrophages in mice treated by combined therapy exhibited a descending trend without statistical significance. Next, we also used Olaparib, another FDA-approved PARP inhibitor, to test the *in vivo* anti-tumor effect of the HDACi-PARPi combination. Olaparib or Entinostat could inhibit tumor growth, but the combination therapy achieved a much better tumor growth inhibition effect (Supplementary Fig. S6E). To demonstrate the crucial role of anti-tumor immunity in the HDACi-PARPi combination, we tested the anti-tumor effects of this combination in the immunodeficient BALB/c-null mice. During the HDACi-PARPi treatment, the immunodeficient BALB/c-null mice did not show a substantial ascites control (**Fig. 6F-G**). In summary, Niraparib combined with Entinostat significantly enhanced the HRD-EXCUTE signature in ascites and established the inflamed-tumor microenvironment, substantially inhibiting tumor growth.

PARPi combined with ICB were applied to treating ovarian cancer in several clinical trials, but had achieved unsatisfactory clinical efficacy. 14 We tried to figure out whether the combination of PARPi, HDACi and ICB could substantially overcome immunotherapeutic resistance in ovarian cancer. The animal experiment scheme was similar to that of the Niraparib-Entinostat combined therapy (Supplementary Fig. S5A). To test whether the efficacy of the low-dose of triple-combined therapy was similar to the double combination, Entinostat and Niraparib in the triple-combined therapy was applied at half dosages. Triple combination therapy further delayed severe ascites occurring (Supplementary Fig. S5B) and substantially repressed tumor growth (Supplementary Fig. S5C-D), compared to the double combination therapy. The triple combination therapy further promoted HRD-EXCUTE signature of ascites cells (Supplementary Fig. S6A) and upregulated the number of immune cells in ascites when compared to the double combination therapy (Supplementary Fig. S6B-M). In details, almost all the cytotoxic cells were significantly upregulated in the triple treatment, compared to the placebo group or *α*-PD-1 group. PRF1^+^CD4^+^T cells and IFNγ^+^CD8^+^T cells in ascites were significantly upregulated in the triple combination group compared to the double combination group. IFNγ^+^CD4^+^T cells, TNFα^+^CD4^+^T cells, TNFα^+^CD8^+^T cells, IFNγ^+^NK cells, PRF1^+^NK cells, M1 macrophages and CD86^+^ DC cells were slightly upregulated in ascites of the triple combination group, compared to the double combination group. Moreover, the low-dose triple combination therapy containing Olaparib, Entinostat, and α-PD-1 could further substantially repress tumor growth (Supplementary Fig. S6F). Thus, Olaparib, similar to Niraparib, could synergistically work with Entinostat to inhibit tumor growth, indicating HDACi-PARPi-based immunotherapy in treating HRD ovarian cancer. In summary, the triple combination of Niraparib/Olaparib, Entinostat and *α*-PD-1 could significantly enhance HRD-EXCUTE signature in ascites and substantially promote the establishment of the inflamed-tumor microenvironment, which would further overcome the immunotherapeutic resistance in HRD ovarian cancer.

## Discussion

In this study, we constructed the HRD functional phenotype HRD-EXCUTE containing 15 upregulated hub genes in HRD ovarian cancer. HRD-EXCUTE substantially prolonged survival in HRD ovarian cancer. Loss of HRD-EXCUTE in HRD patients was intensely related to immunotherapy insensitivity. HRD-EXCUTE was positively associated with immune-inflamed subtypes and negatively correlated with the proliferative, differentiated and mesenchymal subtypes in HRD patients. Furthermore, HRD-EXCUTE could inflame tumor microenvironment by intensifying tumor mutation burden and increasing neoantigens, boosting the infiltration of immune cell into tumor microenvironment. Moreover, the combination of PARPi and HDACi augmented cGAS-STING pathway and promoted histone acetylation around HRD-EXCUTE hub genes, resulting in the enhanced HRD-EXCUTE, which could promote the sensitivity of HRD^+^HRD-EXCUTE^-^ patients to immunotherapy (Supplementary Fig. S8).

HRD is a common feature which occurs in most of tumors, especially in ovarian cancer.^31^ HRD causes tumor genomic instability, which derives considerable aberrant endogenous DNA fragments, activating innate immune system by the cGAS-STING pathway, etc.^4^ Furthermore, HRD is positively correlated with tumor mutation burden and neoantigen load, playing a role in promoting tumor immunogenicity and the infiltration of immune cells into tumor microenvironment.^21^ Consistently, we also found that HRD ovarian cancer was enriched in immune related pathways, especially regarding of the response to chemokine. Thus, HRD is a potent biomarker for immunotherapy. However, ovarian cancer patients seemed unable to benefit from immunotherapies as expected, regardless of approximately 50% of patients harboring HRD. Some HRD patients might have low immunogenicity and inefficient immune response.^16^ In our study, although 15 immune hub genes significantly upregulated in HRD patients, HRD-EXCUTE containing these hub genes was slightly correlated with HRD, of which the correlation coefficient was 0.29. Collectively, HRD was not highly consistent with the inflamed immunophenotype. According to our decision tree, HRD^+^HRD-EXCUTE^+^ patients had more immune-inflamed subtypes while HRD^+^HRD-EXCUTE^-^ and HRD^-^ patients shared a similarity in preferring to be proliferative, differentiated and mesenchymal subtypes. Indeed, HRD^+^HRD-EXCUTE^+^ patients had more substantially prolonged survival and better immune efficacy than other two groups. Together, the lack of HRD functional phenotype might account for immunotherapy inefficiency in HRD ovarian cancer.

Mechanistically, HRD-EXCUTE elevated mutational signatures such as mutSig2, mutSig3 and mutSig7, which could promote tumor mutation burden and neoantigen load. HRD-EXCUTE could further induce the interferon phenotype including the expression of IFNG and PD-1/PD-L1 and the infiltration of immune cells into tumor microenvironment. Furthermore, HRD-EXCUTE could not increase drug sensitivity of HRD cancer cells, but mainly promoted the immunological phenotype in HRD cells. In summary, Loss of HRD-EXCUTE phenotype might substantially attenuate tumor immunogenicity in HRD patients.

So far, clinical trials have found that HRD patients could not substantially benefit from the PARPi-based immunotherapies. Niraparib in combination with pembrolizumab exhibited an ORR of 18% in TOPACIO trial,^14^ which meant that PARPi might not be able to efficiently induce immunogenicity in the HRD patients. We hypothesized that the PARP inhibitor-based combination could significantly facilitate tumor immunogenicity and reverse immunotherapeutic resistance. Here, we applied *CAMP* web to figure out agents that can induce HRD-EXCUTE. As expected, most DNA damage agents and well-studied immunoenhancing agents strongly induced the HRD-EXCUTE, including Entinostat, a highly selective histone deacetylase (HDAC1/3) inhibitor, inhibits the removal of the acetyl groups on histone and is typically related to transcriptional activity.^32^ It was approved that HDACi can not only promote apoptosis and cell cycle arresting in tumor cells, but also modulate the tumor immune microenvironment and strengthen the response to PD-1 inhibition.^33^ Moreover, HDACi combined with PARPi could enhance PARPi-based therapy in ovarian cancer by inhibiting the arrest of cell cycle and damaging HR repair.^34^-^36^ However, limited body of works to date have explored the tumor cell-intrinsic effects of the combination on antitumor immunity. In our study, we found that the HDAC deacetylation was significant activated in HRD patient who lost HRD-EXCUTE. The HDAC inhibitor Entinostat could promote the HRD-EXCUTE, and further enhance the expression levels of HRD-EXCUTE by combining with PARPi. PARPi combined with HDACi could further activate the cGAS-STING pathway and increase the histone acetylation around genomic locus of HRD-EXUTE hub genes. In the HRD ovarian cancer mouse model, Niraparib/Olaparib combined with Entinostat could significantly enhance HRD-EXCUTE signature in ascites and promote the establishment of the inflamed-tumor microenvironment, which substantially inhibited tumor growth. Furthermore, the triple combination of Niraparib/Olaparib, Entinostat and *α*-PD-1 could further overcome the immunotherapeutic resistance in HRD ovarian cancer. Although several previous studies indicated the direct tumor inhibition of the HDACi-PARPi combination, 34-36 we showed the HDACi-PARPi combination exhibited a more anti-tumor effect in the immunocompetent mice than the immunodeficient mice with the same drug dose and frequency. Thus, our animal models revealed the unexpected anti-tumor immunity enhanced by the HDACi-PARPi combination.

In summary, the HRD functional phenotype HRD-EXCUTE was constructed as a potent biomarker for the identification of HRD patients who could benefit from the immunotherapy. HRD patients without HRD-EXCUTE were largely unresponsive to immunotherapy. It is of value to test and optimize the HRD-EXCUTE in the immunotherapeutic cohorts of ovarian cancer to precisely predict the immunotherapeutic efficacy. Furthermore, as shown in our data the combination of PARPi with HDACi could improve the efficacy of the PARPi-based immunotherapy in ovarian cancer by augmenting the HRD functional phenotype. This finding significantly laid a foundation for developing the combination of PARPi and HDACi in the treatment of the HRD ovarian cancer. However, we only investigated the *in vivo* anti-tumor effect of Entinostat combined with PARP inhibitors. Several other HDAC inhibitors, such as Vorinostat and Givinostat, could also promote the HRD-EXCUTE signature in ovarian cancer cell lines (data not shown). Thus, further *in vivo* investigation of other HDACi-PARPi combinations in ovarian cancer is worthwhile.

## Methods

### Data collection and processing

The transcriptomic data and corresponding clinical information were obtained from three Cancer Genome Atlas (TCGA) cohorts (OV, LUSC, CESC). The HRD Scores were obtained from the pan-cancer study.^2^ The expression data of ovarian cancer affy cohort were extracted from Gene Expression Omnibus (GSE18520, GSE30161, GSE19829, GSE63885, GSE26193, GSE9891, GSE14764, GSE23554, GSE26712, GSE31245). The *combat* R package was applied to remove batch effect in affy cohort. The expression data and other omics data of Imvigor210 cohort were obtained from the R package Imvigor210.^17^ The expression data and corresponding data of I-SPY2 trial were acquired from GSE173839.^18^ The drug-induced expression profiles were obtained from CMAP database (https://clue.io/).

### Differential gene expression analysis

*Limma*, *EdgeR*, and *DESeq2* were applied to explore the common differentiated expression of genes (DEGs) between HRD high and low group in ovarian cancer. Based on Benjamini-Hochberg method, false discovery rate (FDR) was corrected by multiple tests of raw *p* values. |log2fold change | > 1 and FDR < 0.01 were set as the threshold for the identification of DEGs.

### Functional enrichment analyses

The *clusterProfiler* R package was applied for gene ontology (GO) annotation biological process enrichment analysis of the DEGs. The parameters used in the *clusterProfiler* R package were set as the default. *p* < 0.05 was set as the threshold of identifying the GO functions of DEGs.

### Identification of hub genes and calculating HRD-EXCUTE

The upregulated genes of DEGs in HRD positive group were analyzed by *STRING* Web, and the *STRING* protein-protein interaction results were further analyzed by *CytoHubba* in the *Cytoscape*. The parameters used in the *STRING* and *CytoHubba* were as the default. The number of hub genes were set to 15. HRD-EXCUTE was defined as the average level of 15 hub genes.

### Decision tree modeling

The survival decision tree was constructed by the *rpart*, *partykit* and *survival* R packages. Overall survival and status were the response factor. HRD Score and HRD-EXCUTE were independent variables, other parameters were the default. The cutoff value of pruning was set for the purpose of maximizing the significance of the tree and simplifying the tree. In other clinical trials, HRD Score was replaced by different HRD assessing methods, for example, FMOne mutation burden per MB in Imvigor210 cohort, and PARPi7 in I-SPY2 trial.

### Immune cells infiltration evaluating

In order to study the enrichment of immune cells, the analysis was conducted by *immunedeconv* implemented with *xCell* algorithm. *xCell* is able to analyze cell type enrichment over 64 types of immune and stroma cells on the basis of gene expression data, which can reduce associations among closely related cell types.^19^ The parameters adopted in the *immunedeconv* - were default parameters.

### Connectivity Map Analysis

The CMAP web, the largest perturbation-driven gene expression dataset over the world, collects a large quantity of drug-induced gene expression profiles.^20^ The *CMAP* web was used to search for drugs which could induce expression profiles similar to HRD-EXCTUTE. HRD-EXCUTE hub genes were entered into Query tool on the *CMAP* web, and the experimentally-examined L1000 sub-database was selected. After querying, drugs with raw connective score > 0.5 and FDR < 0.05 were selected for further analyses.

### ChIP-seq analysis

ChIP-seq data were obtained from GSE131140 and GSE122153 dataset, assessed for quality parameters using *FastQC*, and then trimmed with *Trim_galore*. Reads were aligned to the human reference genome (GRCh38) using *Bowtie* with default options. Reads were dealt by *Samtools* and processed for peak-calling by *MACS2* with default options. The different peaks between BRCA1-null and BRCA1-wt cells were called by R package *Diffbind* and peaks annotation were done by R package *ChIPpeakAnno*. Bigwig files were visualized using *IGV*.

### Cell culture

Ovarian cell lines SKOV3, OVCAR5 and CAOV3 were purchased from ATCC. The normal, immortalized ovarian cell line IOSE80 were also purchased from ATCC. ID8 cell line was a gift from Pro. Xia Xiaojun in our cancer center. All cells were cultured in DMEM supplemented with 7% FBS and antibiotics (50 mg/mL penicillin/streptomycin) and were verified to be free of mycoplasma contamination. Trp53^-/-^Brca2^-/-^-ID8 cells were constructed by transfecting ID8 cells with lentiCRISPRv2-BSD-sgTp53 and lentiCRISPRv2-PURO-sgBrca2virus. Tp53^-/-^Brca2^-/-^ID8-Luc cells were constructed by transfecting Tp53^-/-^Brca2^-/-^ID8 cells with PGF-GFP-LUC virus, and monoclonal cells were sorted out and seeded into 96-well plates by flow cytometric sorting.

### RNA isolation, qRT-PCR and RNA sequencing

Total RNA from cell lines was isolated using EZ-press RNA Purification Kit (EZBioscience), and was converted to cDNA using the HiScript II Q RT SuperMix for qPCR (Vazyme). For the Quantitative Real-time PCR (qRT-PCR), the designed primers were listed in the **Supplementary Table S1**. GAPDH/β-actin was adopted as internal controls. qRT-PCR was performed according to the instructions of ChamQ SYBR qPCR Master Mix (Vazyme) by Roche Applied Science LightCycler 480. The relative expressions of different genes were calculated using the 2^-ΔΔ^*Ct* method relative to GAPDH/β-actin.

We treated the OVCAR5 cancer cell lines with Olaparib (10μM)/Niraparib (10μM) and Entinostat (5μM) for 12 hours and extracted RNA for high-throughput RNA sequencing. Briefly, total RNA was used as input material for the RNA sample preparations. Sequencing libraries were generated using NEBNext Ultra RNA Library Prep Kit for Illumina (NEB, USA, Catalog #: E7530L) following manufacturer's recommendations and index codes were added to attribute sequences to each sample. The qualified libraries were pooled and sequenced on Illumina platforms with PE150 strategy in Novogene Bioinformatics Technology Co., Ltd (Beijing, China), according to effective library concentration and data amount required.

### Western blotting

Cell lines were lysed in RIPA lysis buffer containing protease inhibitor PMSF. Protein samples were loaded in the 10% SDS-PAGE and transferred to polyvinylidene fluoride membranes (Millipore). The membranes were blocked with Tris Buffered Saline containing 0.01% Tween 20 buffer and 5% nonfat milk, and incubated with primary antibody overnight at 4℃, following by secondary antibody incubation for 4 h at room temperature. Antibodies were listed in the **Supplementary Table S2**. After incubation with Clarity Western ECL Substrate (BIO-RAD), protein signals were detected and visualized by Amersham Imager 600 detection system.

### OT-I *in vitro* killing assay

The splenocytes isolated from OT-I mice were activated with 2ng/ml OVA257-264 (N4, Sangon Biotech) and 10 ng/ml IL-2 for 3 days. ID8 cells were pulsed by 1ug/ml N4 peptide for 30 minutes, and activated CD8^+^T cells were co-cultured with N4-pulsed ID8 cells in the killing medium (RPMI 1640, 2% FBS) at the ratios of 1:1. After 4 hours, cells were labelled by anti-CD45.1 and anti-caspase3 for 30mins, and analyzed by flow cytometry to calculate the ratio of dead tumor cells (CD45^-^Caspase3^+^). Cytokines and inhibitors were shown in the **Supplementary Table S3**.

### Macrophage phagocytosis Assay

Bone marrow cells (BM) were harvested from C57BL/6j femurs. BM cells were cultured with RPMI 1640 Medium containing 10% heat-inactivated FCS, 10ng/mL murine IL4 and 20 ng/mL murine M-CSF. On day 5, ID8 cells were pretreated by Niraparib and Entinostat for 24 hours, and followed by CFSE labeling. Then these labeled cells were cocultured with BMDM for 2 hours. CD11b^+^CFSE^+^ cells were defined as the engulfed cells. Cytokines and inhibitors were shown in the **Supplementary Table S3**.

### Animal studies

The 6-week-old female C57BL/6 mice and BALB/c-null mice were purchased from the GuangDong GemPharmatech Co.,Ltd (Guangzhou, China). All animal experiments were conducted in accordance with the institutional guidelines and approved by the Animal Care and Use Committee in our cancer center. To construct *in vivo* models, 5×10^6^ Trp53^-/-^Brca2^-/-^-ID8-Luc cells were intraperitoneally injected into C57BL/6j mice/BALB/c-null mice to build the tumor ascites models. After three weeks, mice were detected by *In vivo* Imaging System (IVIS; PerkinElmer, Inc.) to sort out the tumor-bearing ones which were then randomly divided into groups. Tumor progression was weekly monitored by IVIS. Inhibitors and *α*-PD-1 antibody were shown in the **Supplementary Table S3**.

### Analysis of tumor-infiltrating leukocytes and flow cytometry

In the end of the experiment, mice were euthanized, and the ascites were collected by syringes for further analyses. If ascites quantity was too small to meet experiment requirement, lavage by 1xPBS would be carefully applied in abdominal cavity to collect enough ascites. After wash, TILs were stimulated by PMA (MDbio), Ionomycin (Peprotech) and GolgiStop™ (Biolegend) for 3 hours. After stimulation, ascites cells were incubated with the corresponding antibodies for Flow Cytometry. For intracellular staining, the cells were fixed and permeabilized by the Cytofix/CytoPerm BUF KIT (BD Pharmingenand). Viable cells were labeled by Zombie Aqua™ Fixable Viability Kit (Biolegend). Related antibodies were shown in **Supplementary Table S4**.

### Statistics

All the values were presented as mean ± SEM. To compare differences among groups, *Kruskal-wallis* test and *Wilcoxon* test were adopted. R package *ggstatsplot* (v0.9.3) was used to plot the percentage stacked bar and calculate the statistical significance. The calculation results were displayed as follows *p < 0.05; **p < 0.01, ***p < 0.001, and *n.s*. (no significance). *p* < 0.05 was considered to be statistically significant.

### Data availability statement

The datasets used in the present study are available from the TCGA (https://portal.gdc.cancer.gov/) and the Gene Expression Omnibus (GEO, https://www.ncbi.nlm.nih.gov/geo/). The IMvigor210 dataset is available from the R package *Imvigor210*. The CAMP dataset is available from the CMAP web (https://clue.io/). The script for the classification model will be made available upon request (without restrictions) to the corresponding authors. The bioinformatic and experimental results including RNA sequencing data are submitted on github(https://github.com/Yangd38/HRD-functional-phenotype).

### Code availability statement

The R code for the statistical analyses and for figure generation is available upon request from the authors.

## Supplementary Material

Supplementary figures and tables.

## Abbreviations

HRD: homologous recombination deficiency
HRD-EXCUTE: Transcriptomic functional Phenotype
RPS: Repair Proficiency Score
PARPi7: signature constituted of 7 DNA repair genes
