# Supplementary material for: Loss of HRD functional phenotype impedes immunotherapy and can be reversed by HDAC inhibitor in ovarian cancer
Source: Int J Biol Sci. 2023 Mar 21;19(6):1846–60. doi: 10.7150/ijbs.79654 (PMC10092773; doi:10.7150/ijbs.79654)
Supplement: Supplementary file 1 — Supplementary figures and tables. [file ijbsv19p1846s1.pdf]

Supplementary Data

**SFigure 1.** HRD promotes immune-transcriptomic phenotype in ovarian cancer.
**SFigure 2.** HRD-EXCUTE determined HRD patients' prognosis and immunotherapeutic efficacy.
**SFigure3.** HRD transcriptomic phenotype promotes tumor immunogenicity of ovarian cancer.
**SFigure4.** The combination of HDACi and PARPi greatly promoted HRD-EXCUTE.
**SFigure5.** The combination of HDACi and PARPi greatly promoted HRD-EXCUTE.
**SFigure6.** Triple therapy suppressed HRD ovarian tumor growth in vivo.
**SFigure7.**Triple therapy promoted HRD-EXCUTE and lymphocytes infiltrating in ascites.
**SFigure8.**Model for promoting immunotherapy in HRD patients by inducing HRD-EXCUTE.
**Supplementary Table 1.** Forward and reverse primers for RT-PCR.
**Supplementary Table 2.** Antibodies used for western blotting.
**Supplementary Table 3.** Inhibitors, cytokines and mouse therapeutic antibodies.
**Supplementary Table 4.** Fluorescence labeled-antibodies used for flow cytometry analysis.

Supplementary figures:

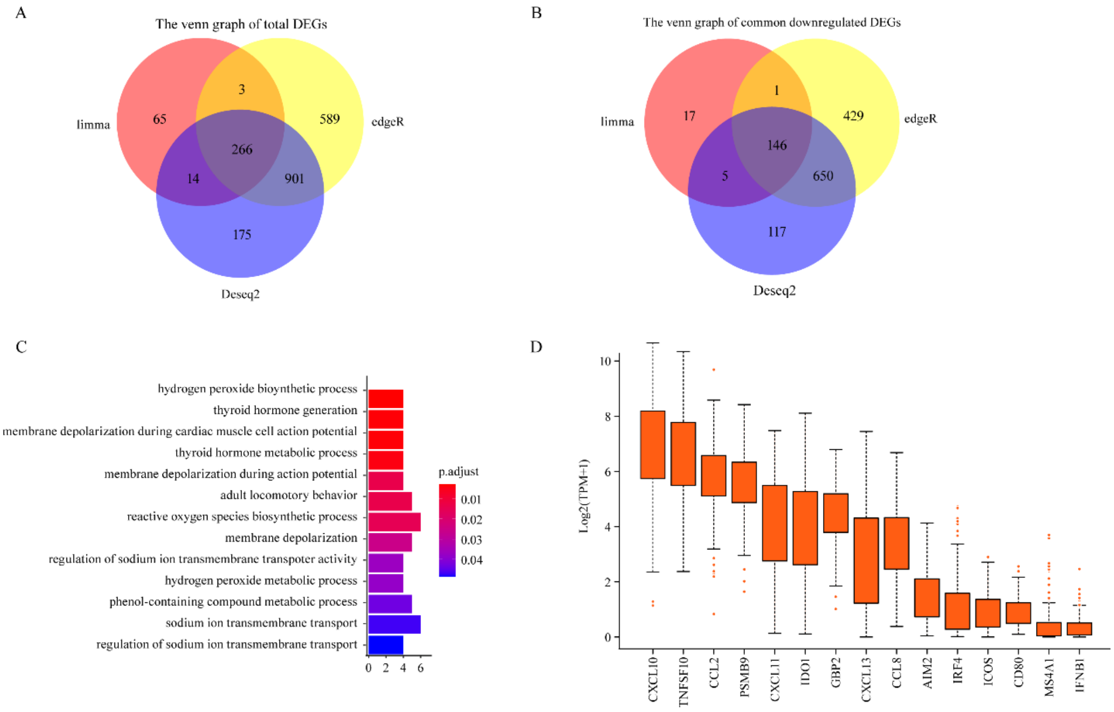

SFigure 1. **HRD promoted immune-transcriptomic phenotype in ovarian cancer**. (A)Venn map of totally differentiated expressions between HRD-high and low group and (B) downregulated genes in HRD-high group among *Limma*, *EdgeR* and *Deseq2* methods. (C) The GO biological process enrichment of downregulated genes in HRD-high group. (D) The expression levels of HRD-

EXCUTE core genes in TCGA-OV.

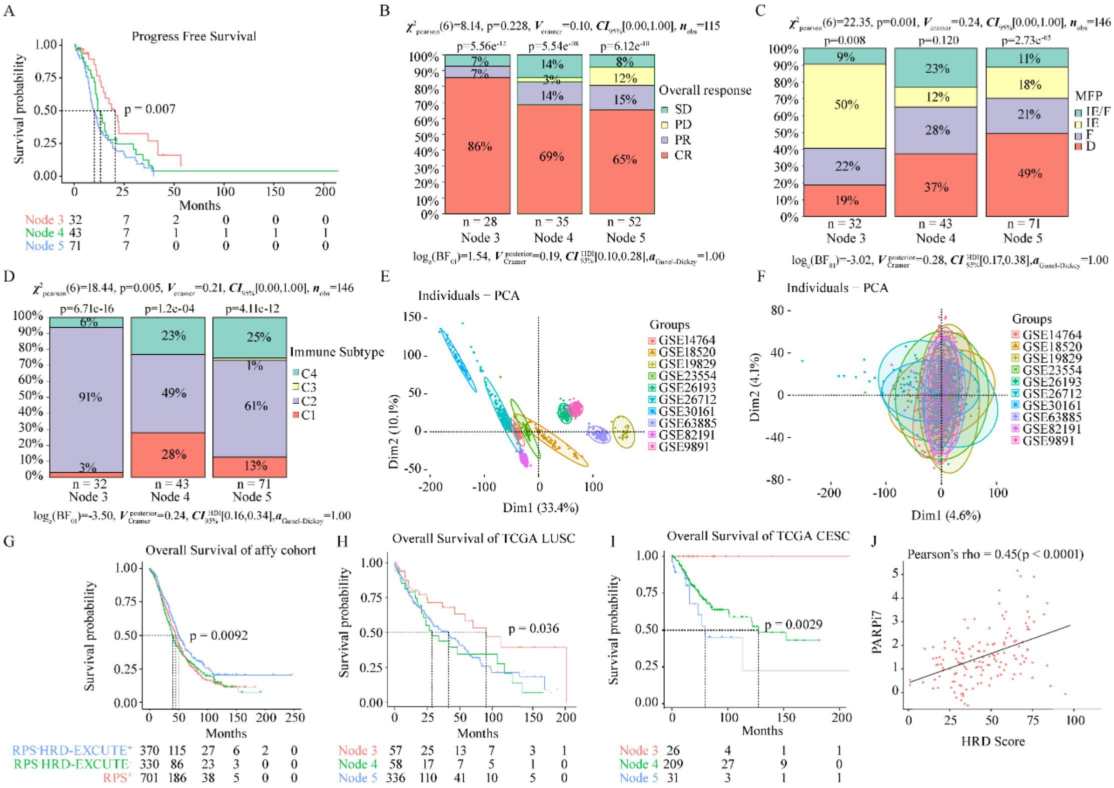

SFigure 2. HRD-EXCUTE determined HRD patients' prognosis and immunotherapeutic efficacy. (A) the progress free survival in three subgroups of the decision tree in TCGA-OV. (B) (B) The distribution of treatment outcomes of the first course among three subgroups of the decision tree in TCGA-OV. The percentage stacked bar plot showed two different microenvironmental subtypes (C) and (D) in TCGA-OV. Principal component analysis plot illustrated the observed batch effect in 10 datasets of affy cohort (E) and mitigation of the batch effect by *combat* algorithm (F). (G) The overall survival plot showed the different prognosis among RPS$^-$HRD-EXCUTE$^+$, RPS$^-$HRD-EXCUTE$^-$, and RPS$^+$ subgroup in the affy cohort. The overall survival plot was showed in the decision tree of HRD and HRD-EXCUTE in (H) TCGA-LUSC and (I) TCGA-CESC. (J) The scatter plot showed the correlation between HRD Score and PARPi7 in the TCGA-OV.

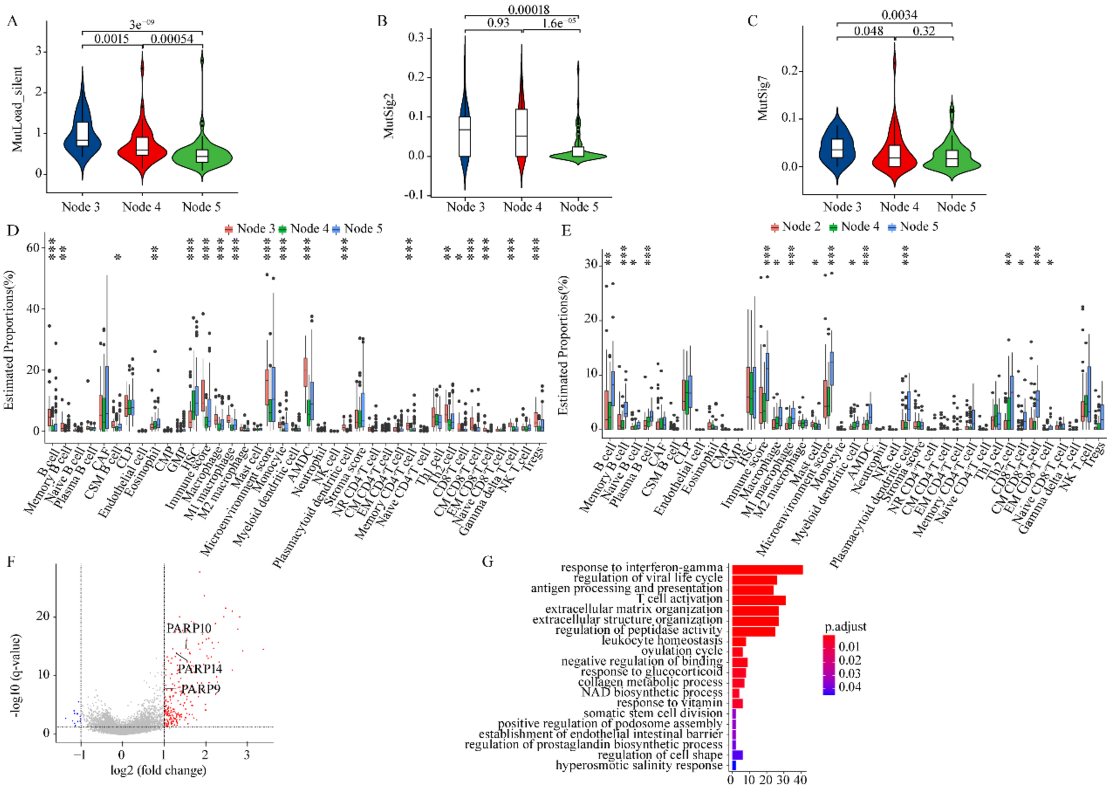

SFigure3. HRD transcriptomic phenotype promoted tumor immunogenicity of ovarian cancer. (A) The silent mutation load per Mb, (B-C) the MutSig2/7 from COSMIC (https://cancer.sanger.ac.uk/cosmic) in the TCGA-OV. The boxplots showed the tumor microenvironmental components in (D) the IMvigor210 cohort and (E) the I-SPY2 trial. The microenvironmental components were estimated by *xCell* algorithm. (F) The volcano plot showed the differential genes between DI and DnI subgroups of the CCLE cell lines. (G) The bar plot showed the upregulated pathways in DI subgroups of the CCLE cell lines.

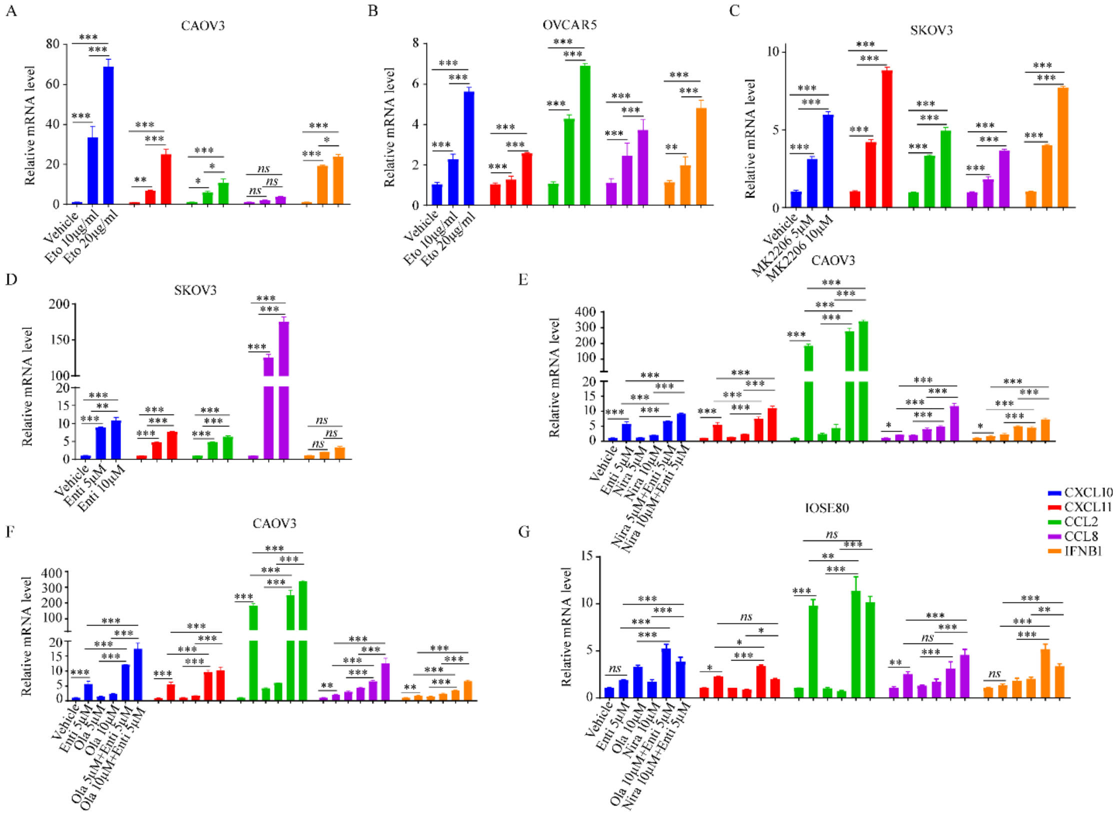

SFigure4. The combination of HDACi and PARPi greatly promoted HRD-EXCUTE. The relative expression levels of CXCL10/11, CCL2/8 and IFNB1 were shown in A-F. (A)CAOV3, (B) OVCAR5 cells were treated by Etoposide for 12 hours, and SKOV3 cells were treated with MK2206 (C) and Entinostat (D) for 12 hours. CAOV3 cells were treated with Niraparib (E), Olaparib (F) and Entinostat for 12 hours, (G) IOSE80 cells were treated with Niraparib /Olaparib and Entinostat for 12 hours, followed by qPCR to examine the expression levels of HRD-EXCUTE hub genes.

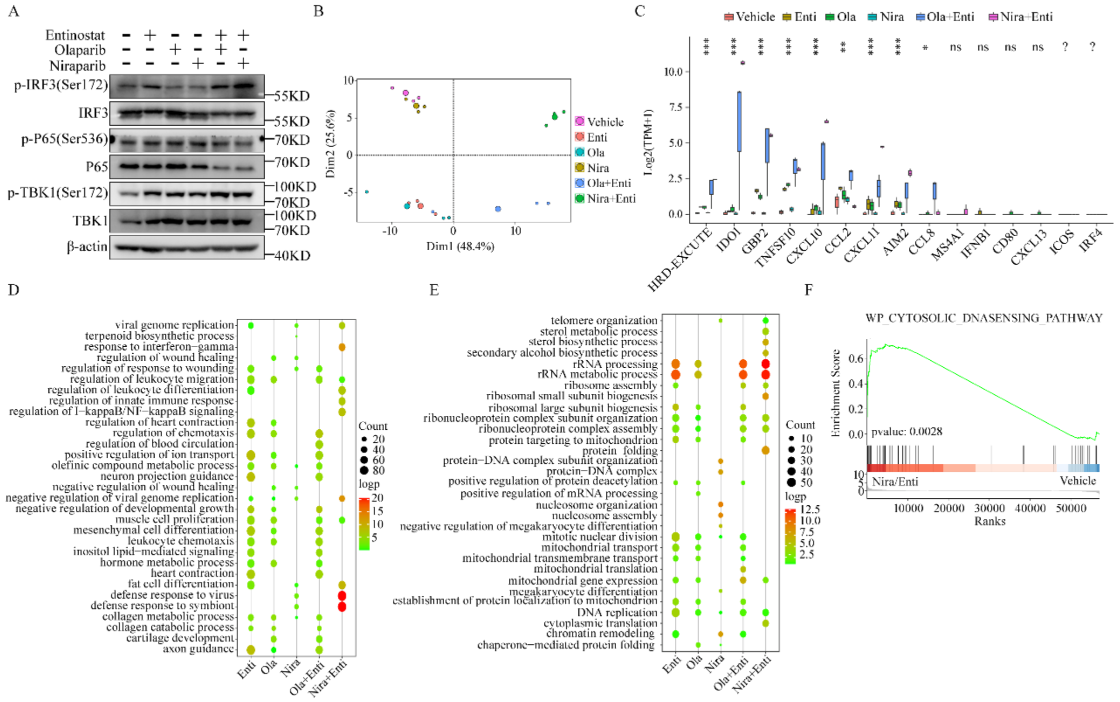

SFigure5. The combination of HDACi and PARPi greatly promoted HRD-EXCUTE. (A)

IOSE80 cells were treated by Olaparib (10μM)/Niraparib (10μM) and Entinostat (5μM) for 12 hours, followed by Western Blotting to examine the phosphorylation of TBK1, IRF3 and P65. (B) The Principal Component Analysis of RNA sequencing performed by R package *factoextra* and *FactoMineR*. (C) The boxplots showed the expression levels of the hub genes in the HRD-EXCUTE in the PARPi-HDACi treatment. The functional enrichments of the biological process were performed by the R package *clusterProfiler* in (D) the significantly upregulated genes and (E) the significantly downregulated genes of experimental subgroups relative to the vehicle subgroup. (F) The R package *clusterProfiler* performed gene Set Enrichment Analysis (GSEA) in the significantly upregulated genes of the Niraparib-Entinostat subgroup relative to the vehicle subgroup.

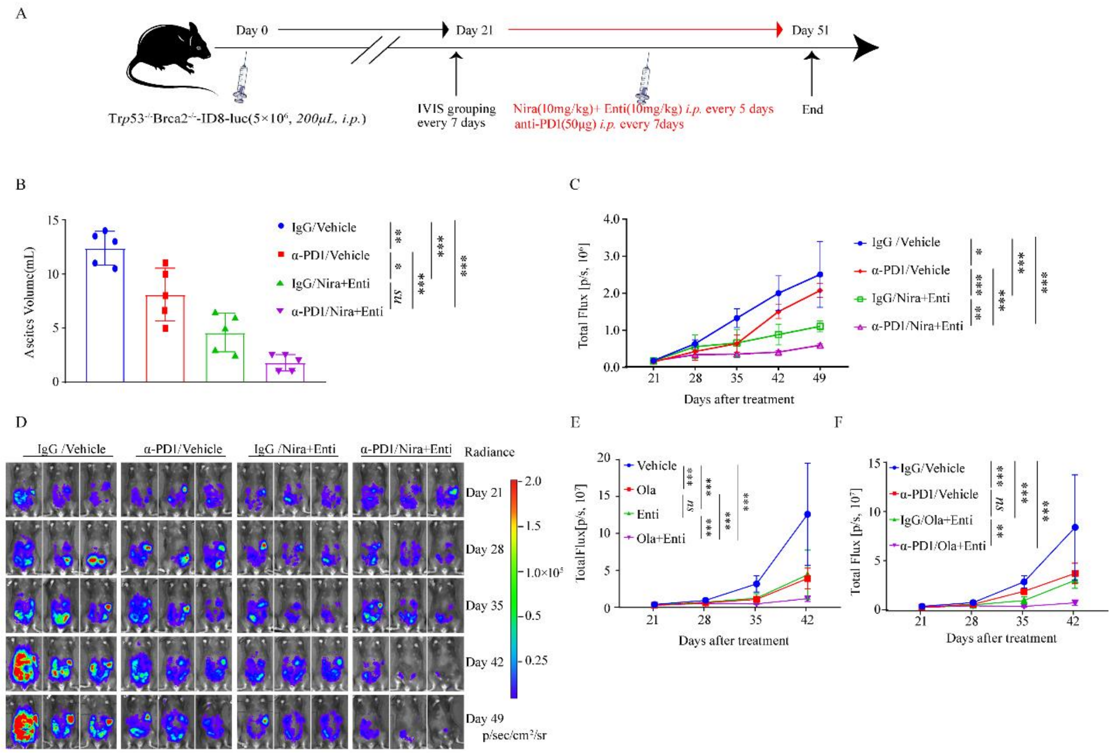

SFigure6. Triple combination suppressed HRD ovarian tumor growth in vivo. (A) The processes of animal experiment were displayed in the diagram. (B) Bar plot showed ascites volume (ml) in four subgroups by the end of experiment. (C) Tumor burden was evaluated by quantifying total flux with PE Living Image software. Data were presented by mean value of 5 mice per group ± standard error. (D) Bioluminescence of C57BL/6j mice after inoculation of Trp53$^{-/-}$Brca2$^{-/-}$-ID8-luc cells. (E-F) The C57BL/6j mice were treated with Olaparib-Entinostat combination and Olaparib-Entinostat-α-PD1 combination. Tumor burden was evaluated by quantifying total flux with PE Living Image software. Data were presented by mean value of 5 mice per group ± standard error. Nira: Niraparib, Ola: Olaparib, Enti: Entinostat.

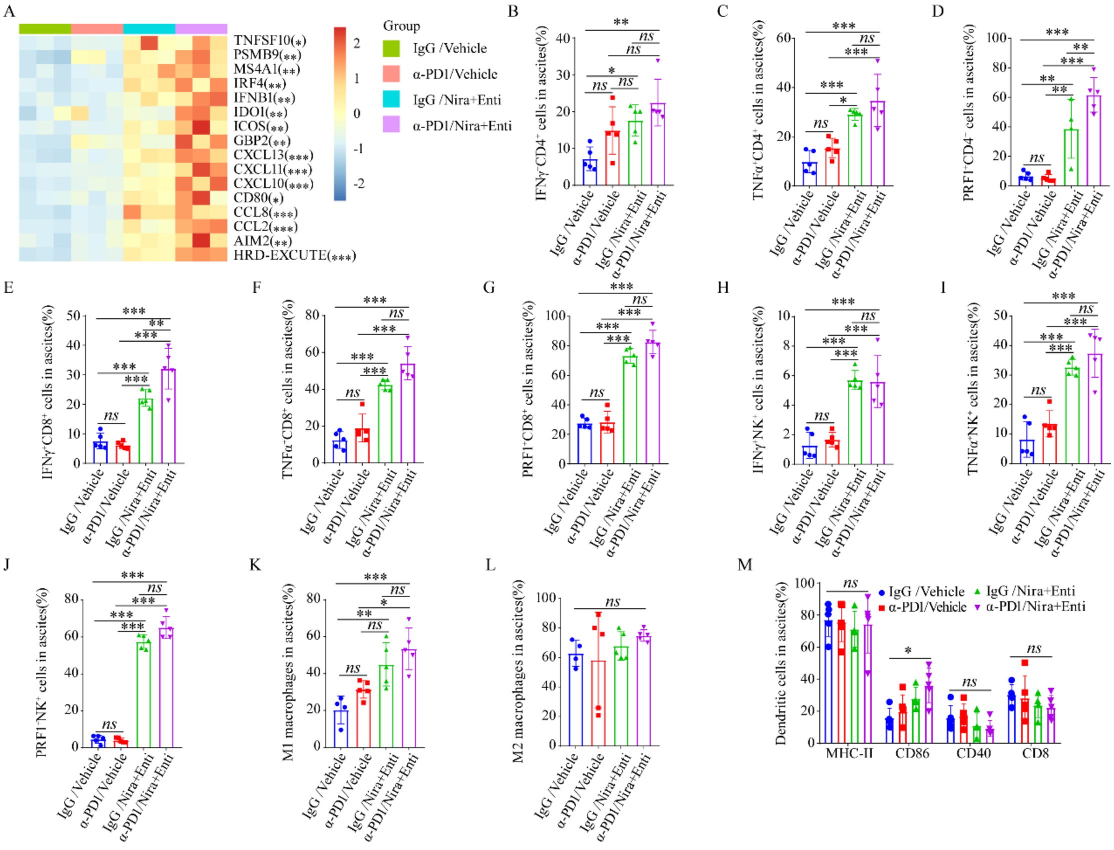

SFigure7. Triple combination promoted HRD-EXCUTE and lymphocytes infiltrating in ascites. (A) Heatmap showed the relative expression levels of HRD-EXCUTE hub genes in ascites cells. Boxplots showed the fraction of immune cells: (B) IFNγ$^+$CD4$^+$T cells, (C) TNFα$^+$CD4$^+$T cells, (D) PRF1$^+$CD4$^+$T cells, (E) IFNγ$^+$CD8$^+$T cells, (F) TNFα$^+$CD8$^+$T cells, (G) PRF1$^+$CD8$^+$T cells, (H) IFNγ$^+$NK cells, (I) TNFα$^+$NK cells, (J) PRF1$^+$NK cells, (K) M1 macrophages, (L) M2 macrophages and (M) DC cells. Nira: Niraparib, Ola: Olaparib, Enti: Entinostat.

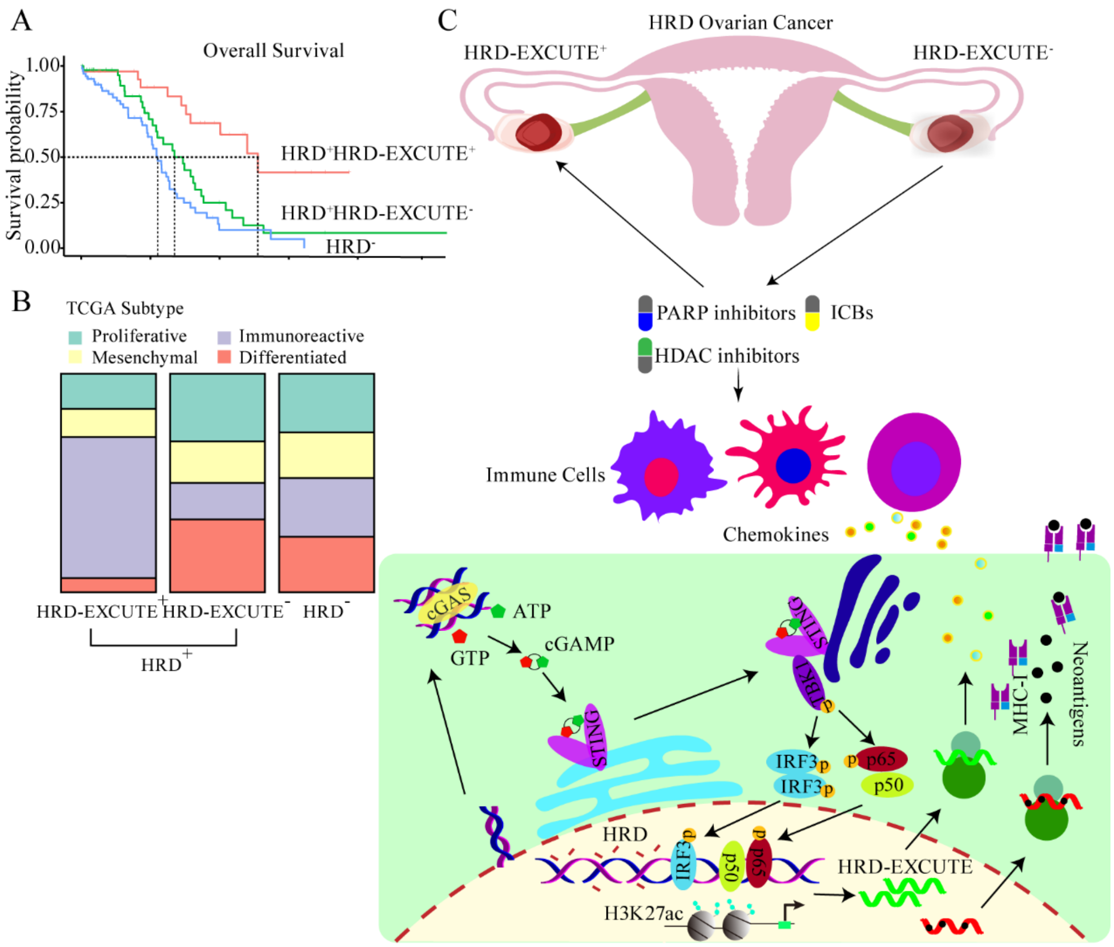

SFigure8.Model for promoting immunotherapy in HRD patients by inducing HRD-EXCUTE.

The HRD functional phenotype HRD-EXCUTE substantially prolonged (A) the overall survival and was positively correlated with (B) the immune-inflamed subtype in HRD ovarian cancer. (C) Mechanistically, in the HRD^{+}HRD-$EXCUTE^{+}$ patients, cytosol DNA caused by HRD promoted substantially intensified tumor mutational burden and increased neoantigens, which in turn attracted immune cells to migrate into tumor tissues and kill tumor cells. PARPi combined with HDACi enhanced HRD-EXCUTE by augmenting cGAS-STING pathway and promoting histone acetylation around HRD-EXCUTE hub genes, which could also promote the sensitivity of HRD^{+}HRD-$EXCUTE^{-}$ patients to immunotherapy.

Supplementary Table 1. Forward and reverse primers for RT-PCR.

Name	Sequence
m-b-actin-F	GGCTGTATTCCCCTCCATCG
m-b-actin-R	CCAGTTGGTAACAATGCCATGT
m-CXCL10-F	ATGACGGGCCAGTGAGAATG
m-CXCL10-R	GAGGCTCTCTGCTGTCCATC

m-CXCL11-F	TGGAACATGCAGCCACGTAT
m-CXCL11-R	CACTGGTCCGGATTGCAGTA
m-Cd80-F	CATCAAAGCTGACTTCTCTACCC
m-Cd80-R	GGGTTTTTCCCAGGTGAAGT
m-IFNB1-F	CCCTATGGAGATGACGGAGA
m-IFNB1-R	CTGTCTGCTGGTGGAGTTCA
m-CXCL13-F	TTGTGATCTGGACCAAGATGAA
m-CXCL13-R	GACTTTTGCTTTGGACATGTCT
m-CCL2-F	AGATGCAGTTAACGCCCCAC
m-CCL2-R	GCTGAAGACCTTAGGGCAGA
m-CCL8-F	AAGCTGAAGATCCCCCTTCG
m-CCL8-R	GGATGAGAAAACACGCAGCC
m-TNFSF10-F	CCTCAGAAAGTGGCAGCTCA
m-TNFSF10-R	CAGTCCGTACTCGGCATCTC
m-IDO1-F	AGATGTGGGCTTTGCTCTACC
m-IDO1-R	GCAGCACCTTTCGAACATCG
m-IRF4-F	TGGAGGGATTATGCCCCTGA
m-IRF4-R	TGCTTGGCTCAATGGGGATT
m-GBP2-F	CCTGACCAGAGTGGGGTAGA
m-GBP2-R	CAGTCGCGGCTCATTAAAGC
m-AIM2-F	GCCGCCATGCTTCCTTAACT
m-AIM2-R	CTGTCTTGTTCCCACTGCCT
m-PSMB9-F	CCACACCGGGACAACCATC
m-PSMB9-R	TCAGCTATGGCTTGGGCATC
m-MS4A1-F	TTGGCCCTTAAGCCTTGGAG
m-MS4A1-R	TCATGATTTGGACAGCCCCC
m-ICOS-F	TACATGTTCATGGCGGCAGT
m-ICOS-R	ATCAAGTCAGGTGCCCTGTG
h-GAPDH-F	CGCTGAGTACGTCGTGGAGTC

h-GAPDH-R	GCTGATGATCTTGAGGCTGTTGTC
h-CXCL10-F	TGCCATTCTGATTTGCTGCC
h-CXCL10-R	TGCAGGTACAGCGTACAGTT
h-CXCL11-F	AAGGACAACGATGCCTAAATCCC
h-CXCL11-R	CAGATGCTCTTTTCCAGGACTTC
h-CD80-F	GGGAAATGTCGCCTCTCTGAAG
h-CD80-R	CCCAAGTAAGACCAGGGCAC
h-IFNB1-F	TCAAAGTTCATCCTGTCCTTGA
h-IFNB1-R	TCCACTACAGCTCTTTCCATGA
h-CXCL13-F	GCTTGAGGTGTAGATGTGTCC
h-CXCL13-R	CCCACGGGGCAAGATTTGAA
h-CCL2-F	CAGCCAGATGCAATCAATGCC
h-CCL2-R	TGGAATCCTGAACCCACTTCT
h-CCL8-F	CCCAGGTGCAGTGTGACATTA
h-CCL8-R	GGGAGGACCCCACAACACTA
h-TNFSF10-F	GCTGCCTGGCTGACTTACA
h-TNFSF10-R	AAGCAATGCCACTTTTGGAG
h-IDO1-F	GGGCTTTGCTCTGCCAAATC
h-IDO1-R	ATGACCTTTGCCCCACACAT
h-IRF4-F	CTACACCATGACAACGCCTTACC
h-IRF4-R	GGCTGATCCGGGACGTAGT
h-GBP2-F	ATTGTGGGCCTCTATCGCAC
h-GBP2-R	CCAGGTGAGGAGTTTGCCTT
h-AIM2-F	TGGCAAAACGTCTTCAGGAGG
h-AIM2-R	AGCTTGACTTAGTGGCTTTGG
h-PSMB9-F	CTGGGACCAACGTGAAGG
h-PSMB9-R	ATGGCCAGAGCAATAGCG
h-MS4A1-F	ATTCTGTGGGGAAGAGACTGAC
h-MS4A1-R	CTGCCGGGAAAGTCCCATTT

h-ICOS-F	TTGAACACTGAACGCGAGGA
h-ICOS-R	TCGCAGAGTATTTGCCCCC

Supplementary Table 2. Antibodies used for western blotting.

Name	Lot.	Company
Anti-β-actin	66009-1-Ig	Proteintech
Anti-TBK1	28397-1-AP	Proteintech
Anti-p-TBK1 (Ser172)	5483S	Cell Signaling Technology
Anti-IRF3	11312-1-AP	Proteintech
Anti-p-IRF3 (Ser386)	37829S	Cell Signaling Technology
Anti-P65	sc-8008	Santa Cruz Biotechnology
Anti-p-P65 (Ser536)	sc-136548	Santa Cruz Biotechnology
HRP-conjugated Goat Anti-Mouse IgG(H+L)	SA00001-1	Proteintech
HRP-conjugated Goat Anti-Rabbit IgG(H+L)	SA00001-2	Proteintech

Supplementary Table 3. Inhibitors, cytokines and mouse therapeutic antibodies.

Name	Lot.	Company
Niraparib	T3231	TargetMol
Olaparib	T3015	TargetMol
Entinostat	T6233	TargetMol
Etoposide	T0132	TargetMol
MK2206	T1952	TargetMol
Murine-IL2	212-12-50	PeproTech
OVA peptide	T508495	Sangon Biotech
Murine-IL4	CX03	NovoProtein
Murine-M-CSF	CB34	NovoProtein
Murine-α-PD1	BP0146	Bioxcell
Murine-IgG2α	BP0089	Bioxcell

Supplementary Table 4. Fluorescence labeled-antibodies used for flow cytometry analysis.

Name	Lot.	Company
PE/Cyanine7 anti-mouse CD49b	No.108922	Biolegend
PE anti-mouse Perforin	No.154306	Biolegend
APC/Cyanine7 anti-mouse CD45	No.103116	Biolegend
FITC Anti-Active Caspase-3	No.559341	BD Pharmingenand
Brilliant Violet 785™ anti-mouse TNF-α	No.48-8896-42	ThermoFisher

Alexa Fluor® 700 anti-mouse CD4	No.100430	Biolegend
PerCP/Cy5.5 anti-mouse CD8a	No.100733	Biolegend
Ms IFN-Gma Alexa 647 XMG1.2	No.557735	BD Pharmingenand
Zombie Aqua™ Fixable Viability Kit	No.423102	Biolegend
APC/Cy7 anti-mouse CD45	No.103116	Biolegend
CFSE	No.HY-D0938	MedChemExpress
PE anti-mouse F4/80	No.123110	Biolegend
PerCP/Cyanine5.5 anti-mouse CD11b	No.101228	Biolegend
Alexa Fluor® 647 anti-mouse CD206	No.141712	Biolegend
PE/Cyanine7 anti-mouse CD86	No.105014	Biolegend
APC/Cyanine7 anti-mouse Ly-6G/Ly-6C (Gr-1)	No.108424	Biolegend
Pacific Blue™ anti-mouse CD11c	No.117322	Biolegend
APC MHC Class II I-Ad Mono Antibody	No.17-5323-82	eBioscience
PE anti-mouse CD40	No.157506	Biolegend
Fixable Viability Stain 700	No.564997	BD Bioscience
